# Towards a universal concept of vulnerability: Broadening the evidence from the elderly to perinatal health using a Delphi approach

**DOI:** 10.1371/journal.pone.0212633

**Published:** 2019-02-20

**Authors:** Nynke de Groot, Gouke J. Bonsel, Erwin Birnie, Nicole B. Valentine

**Affiliations:** 1 Maternity Care Academic Research Group, Department of Obstetrics and Gynecology, University Medical Center Utrecht, Utrecht University, Utrecht, The Netherlands; 2 Department of Obstetrics and Gynecology/Division of Obstetrics and Prenatal Medicine, Erasmus Medical Center, Rotterdam, The Netherlands; 3 Department of Public Health, Erasmus Medical Center, Rotterdam, The Netherlands; 4 Erasmus School of Health Policy and Management, Erasmus University Rotterdam, The Netherlands; 5 Public Health, Environmental and Social Determinants of Health Department (PHE), World Health Organization, Geneva, Switzerland; Universidad del Desarrollo, CHILE

## Abstract

**Background:**

The concept 'vulnerability' is prevalent in the public domain, health care, social institutions and multidisciplinary research. Conceptual heterogeneity is present, hampering the creation of a common evidence-base of research achievements and successful policies. Recently an international expert group combined a specific literature review with a 2-stage Delphi procedure, arriving at a seemingly universal concept of vulnerability for the elderly with applications for research instruments. We replicated and extended this study, to generalize this result to health in general, and perinatal health in particular.

**Methods:**

Two independent expert panels (general health, perinatal health) repeated the Delphi-procedure, using an extended and updated literature review to derive statements on the concept and defining pathways of vulnerability. Additional views were collected on research tools. Consensus-by-design was explicitly avoided. Data collection and processing was independent.

**Results:**

Both panels showed surprising convergence on the pathways of vulnerability to health/ill-health, and their interaction. The agreed conceptual model describes a dynamic relation between health and ill-health and vulnerability. The 2 key pathways that link to vulnerability, are complementary, but not symmetrical as biological processes of maintaining health or obtaining better health are not reciprocal to recovery, so also not in terms of vulnerability impacts. An individual's degree of vulnerability is the net balance of risk effects and protective and healing factors (socially, biologically and in terms of health literacy and health care access). These factors can for measurement purposes (according to the panels: interview for exploration, checklists for population research) be grouped into ‘material resources’, ‘taking responsibility for one’s own health’, ‘risky activities and behaviors’, and ‘social support’.

Supportive and transforming action can thus be undertaken.

**Conclusion:**

A universal concept of vulnerability in the context of health was successfully derived after careful replication and extension of an international Delphi study on vulnerability among the elderly.

## Introduction

In the past decade there has been an increased focus on “vulnerability” in health [1]. Accepting some heterogeneity of definitions and contexts, it is generally accepted that the population's health, access to and results from medical care are at the individual and aggregate level strongly related to vulnerability [2–4]. Vulnerability, also referred to as social disadvantage or deprivation (these terms are used interchangeably), is thus closely related to the societal challenge of health inequalities. While the existing heterogeneity of vulnerability definitions may be acceptable for the general picture, and advocacy, it is a persistent obstacle for evidence-based action to address it [5]. Several authors proposed a definition of vulnerability [1] with different views of vulnerability as either a determinant of ill-health or impeding factor in the process of recovering from ill-health. Due to this conceptual heterogeneity, research results in this context and recommendations are difficult to compare, lessons learnt in one clinical area or subpopulation cannot easily be transferred to another, altogether hampering the creation of an evidence-base. To our knowledge, the first to converge on an encompassing, global concept of vulnerability was the Commission on Social Determinants (CSDH) of the World Health Organization (WHO) [6] while working on health care and cross-sectoral recommendations to reduce the effects of vulnerability. A formal definition, however, was not outlined.

The starting point of this paper was our engagement in a nationwide evidence-driven reform of perinatal care in the Netherlands, with a focus on the decrease of perinatal health inequalities [7–8]. Perinatal health inequalities are reckoned among the most important inequalities from a public health point of view [9]. Initial differences in perinatal health influence the remaining life span [10–12]: suboptimal physical and cognitive development arises from the two most common adverse outcomes, small for gestational age (SGA) fetuses and prematurity. Both morbidities universally show strong inequality gaps [7, 9, 13–14]. Resulting from intricate genetic-environment (gen-environment) interactions, these inequalities tend to give rise to adult diseases and to similar health problems in the next generation, creating pseudo-hereditary effects of vulnerability and deprivation [15].

We observed, like other researchers of the elderly and the mentally ill [16–17], that lack of vulnerability definitions and measurement tools limits scientific progress in perinatal research and health service practices (e.g. uniform antenatal check at booking visit, and report of inequalities in quality/benchmark documents). Also hindered is coordination with adjacent fields of social policy to address health inequalities, e.g. youth care and regional and city programs for the deprived e.g. directed to deprived or vulnerable young mothers.

Our study aim was to develop a universal, encompassing, concept of vulnerability, which could be applied to perinatal health. To this end, and with consent of the original authors, we adopted and broadened the approach of a similar study focused on vulnerability in the elderly and designed a 2-stage Delphi study [16]. Two independent expert-panels from a wide range of fields, where vulnerability is a key concept, were involved. In the first stage information was formally elicited on how these experts understand and translate 'vulnerability' into care practice, into research concepts, and into measurement approaches. In the second stage it was tested whether the expressed views could be unified into a single concept, or–in the worst case–whether such a unification should be rejected. This Delphi procedure was independently repeated in a special interest group concerned with clinical perinatal health care only. A unified concept of vulnerability, which extends to perinatal health would improve research on perinatal health inequalities, and also contribute to the broader challenge of exchanging and accumulating evidence to address vulnerability and health inequalities in general.

## Methods

### Design

**Fig 1:**
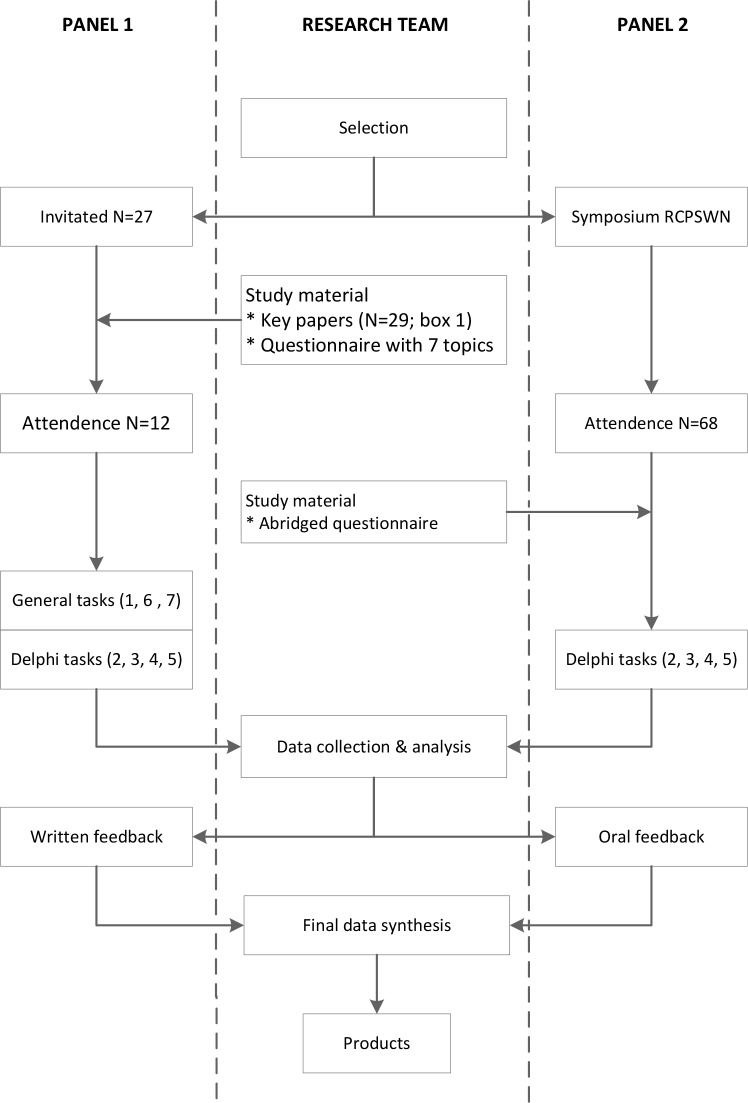
Flowchart of the study design for both expert panels and the research team.

This study uses a two-stage Delphi design [18–19]; a flowchart of the study design can be found in Fig 1.

The first stage consisted of conducting two formal literature searches, creating a reader thereof, and preparing a targeted questionnaire (S1 and S2 Appendices). The questionnaire contained 7 sets of questions (indicated by 'tasks'), to be used in conjunction with the reader. The materials were designed to explore the commonality of different vulnerability concepts (if any), and to explore the potential for an overarching relational scheme.

In the second stage, 2 face-to-face expert meetings were held, involving two entirely different panels of experts. Panel 1 was the general panel consisting of various clinical and public health experts. Panel 2 was the specific panel on vulnerability in pregnancy. Sociodemographic and educational data were collected for descriptive analysis. Panel 1 was offered the reader and questionnaire in advance; the results were discussed during the expert meeting.

Panel 2 did not receive the reader in advance; and instead we presented the questionnaire during a member-meeting of the Regional Perinatal Consortium South-West Netherlands (RCPSWN); every question was preceded by a short oral introduction with abstracted information from the reader. The RCPSWN aims to improve the system of care for vulnerable pregnant women within the South-West Netherlands. As such, panel 2 participants were assumed to be above average informed on perinatal vulnerability allowing for this procedure.

From here on, the interactive Delphi-part of both expert panel meetings was similar. Individual responses to the tasks during the first part of the meeting were grouped by the research group and analyzed for consensus or dissenting views; this allowed ex post the face-to-face comparison of the opinions of the general versus the perinatal panel. The panel results were presented back to both panels separately, for final judgment. At no time was there any information exchanged between these group members. This paper synthesizes the unchanged contents of the agreed panel meeting reports.

### Stage 1

#### Literature extraction for the reader sent to panel 1

An extensive literature search was conducted in Embase May 2014 on the concept of vulnerability and its application in pregnancy. Because the vulnerability literature is poorly structured, where vulnerability is indicated by different terms (e.g. ‘frailty’, ‘disadvantaged’) and content differs according to author (e.g. emphasis on biological factors, access to health care), a formal approach with predefined key words (only), as in a comprehensive review, was not applicable.

The ill-structured evidence required the use of Embase, rather than the conventional databases (PubMed, Medline). Embase covers 98% of PubMed and 100% of MedLine, and beyond these contains more scientific papers in the multidisciplinary fields.

Technically, the authors constructed a number of search blocks, each block containing a set of topic related keywords (S3 Appendix). The blocks were derived from work of the WHO health equity group [20]. The search block ‘pregnancy’ was added as a specifier for vulnerability in the perinatal context. All journals and papers in Embase were deemed eligible for inclusion; no restrictions were made based on journal ranking or paper type.

The initial result (general and perinatal combined) yielded 2,462 unique papers.

To be retrieved and read in full (criteria set and selection made by first and second author independently), a paper had to include a conceptual model of vulnerability OR had to describe a pathway through which vulnerability (however defined, but measurable/operational) affects a measurable outcome, either health- OR health care related.

The paper had to report on a study carried out in developed countries, be written in English or Dutch (the reviewer’s native language) and be available in full text. For the perinatal context, papers had to report on singleton pregnancies and outcomes were restricted to prematurity and small-for-gestational-age. These criteria led to the selection of 148 papers that were retrieved in full. The selection criteria and process were documented. Screening reference lists of selected papers yielded another 84 papers, bringing the total to 232 papers. A summary of the literature review and selection of papers is presented in Table 1.

**Table 1:** Literature review and selection of papers.

Selection of key papers
Construction of search blocks with keywords relating to: generic vulnerability, generic pregnancy, health outcome, pregnancy outcome, PROGRESS*[21].
**Literature review 1: Vulnerability**
Initial results N = 1.548 papers
Screening title and abstract N = 98 papers
Retrieved in full text N = 79 papers
**Literature review 2: Vulnerability in pregnancy**
Initial results N = 914 papers
Screening title and abstract N = 103 papers
Retrieved in full text N = 69 papers
**Reference list screening**
Initial results N = 83 papers
Retrieved in full text N = 70 papers

* PROGRESS: mnemonic for Place of Residence, Religion, Occupation, Gender, Race/Ethnicity, Education, Social status, Socio-economic status

The selected papers were sorted according to their relevance (based on content only; no international guideline available) and ranked 1 through 4. Rank 1 was assigned to papers that either included a full theoretical model of vulnerability, or explored causal pathway(s) from vulnerability to changed health status. Rank 2 was assigned to papers that explored the concept of vulnerability, but without explicitly mentioning a mechanism or by using a term that loosely connects to vulnerability (e.g. lack of uptake of preventive services in an underserved population [which points to a mechanism]). Ranks 3 and 4 were assigned to papers that were either insufficiently relevant for our purpose (e.g. health care needs of vulnerable clients with cancer) or which did not include vulnerability information despite its title. The papers rated the most important, i.e. ranks 1 and 2 (n = 29), were then printed and presented in one reader (for the list of finally 29 included papers, see S1 Table).

#### Pen and paper questionnaire (both panels)

The selected papers provided the formal input for the questionnaire, apart from the expert's pre-existing knowledge. The questionnaire was divided into 7 parts ('tasks'), each containing 1 or more questions covering, among other, key elements of vulnerability, existing definitions and conceptual models of vulnerability and ethical considerations. Table 2 lists the 7 tasks of the pen and paper questionnaire. The response mode was variable and suited to the type of question. For this paper the responses to tasks 2 through 5, reported on by both Panel 1 and 2, are discussed in full since they were most pertinent to deriving a common concept for vulnerability and an associated uniform measurement approach for diagnosing vulnerability in routine care.

**Table 2:** The 7 tasks of the pen and paper questionnaire on vulnerability.

	Questionnaire topics	Panel 1 (general vulnerability)	Panel 2 (perinatal focus)
**1**	Differences and overlap vulnerability concepts	+	-
**2**	Elements of vulnerability	+	+
**3**	Existing definitions of vulnerability	+	+
**4**	Competing models of vulnerability	+	+
**5**	Diagnosing vulnerability in routine care	+	+
**6**	Ethical aspects of vulnerability	+	-
**7**	Hypothetical case reports: vulnerable or not	+	-

+ Topics filled out by panel members;—Topics not filled out by panel members.

The first topic to be discussed (task 2), related to the elements or components of vulnerability. The question listed 29 key elements of vulnerability (for example ‘lack of resources’ and ‘substance abuse’ [See S2 Table for full listing]) that were frequently mentioned in the literature review. Each element was assigned by the research team to one of five, pre-defined domains according to the type of element (‘resources’ [R], ‘coping’ [C], ‘exposure/etiological factor’ [E], ‘manifestation’ [M] and ‘self-efficacy’ [SE]). The experts were invited to assign a score of 0, 1 or 2 to each element according to its importance for assessing a client’s degree of vulnerability during routine care. For each of the 29 elements, a sum score across experts was then calculated to indicate average importance according to the experts.

The next task (task 3) contained a list of 24 published definitions of vulnerability. To each definition, a score of 0, 1 or 2 had to be assigned according to how well (2 = best) it matched the expert’s opinion on vulnerability (for a full list of definitions, see S3 Table). Each expert was required to use all 3 score options 8 times, on the expert level there were 8 ‘best’ definitions, the 8 ‘less good’ definitions and 8 ‘poorest’ definitions. The scores were aggregated for each definition of vulnerability separately (theoretical range: 0–24). Definitions could thus be ranked.

The third task (task 4) presented 5 competing conceptual models of vulnerability to respondents. An important area of disagreement was the focus on vulnerability as a cause of ill health vs. the focus on vulnerability as impeding factor in the recovery process, once being ill. Panel members were asked to indicate the model that, according to their opinion, best described the causal model of vulnerability and health outcomes. Modifications were allowed to the extent that experts could devise a model of their own. The most frequently selected model was regarded as preferred option for the panel meeting.

The last topic (task 5) asked for the ‘best practice’—according to the expert—to assess a client’s degree of vulnerability under routine health service conditions; this was asked for several health care domains (mental health care, the elderly, pregnant women, child and youth care, chronically ill). Panel members were presented—as point of departure—the following options: 1) by checklist (with or without professional support), 2) during an interview or 3) by using registry data [i.e. medical data]. Other methods could be put forward. Experts were invited to write down an example of such a ‘best practice’, if available. For each domain of health care, the distribution of assessment preferences was calculated.

### Stage 2

#### Recruitment and selection

For panel 1, a total of 26 national experts were approached for participation (no reward). Inclusion criteria for experts (set by the second author) were: 1) researchers with a proven track record on vulnerability or vulnerability related topics (e.g. geriatrics [frailty], epidemiology and public health) or clinical specialists with a documented special interest in vulnerable clients (documented in terms of professional position and/or scientific contributions), and 2) they had to have a senior position in their respective organizations (such as head of department/municipal service or associate professor). By selecting experts from both public health and clinical expertise, we aimed to assure the creation of an eclectic comprehensive view on vulnerability. Sociodemographic characteristics of panel members are listed in the middle column of Table 3. Ten experts did not participate in the study because they felt unable to contribute sufficiently. The 13 experts that agreed to participate were sent the reader and questionnaire. Ten experts returned their filled-out questionnaires before the meeting, two handed them in during the meeting. All but one expert were present during the meeting. Panel 1 meeting took place on November 11^th^, 2014 at a central location in The Netherlands (Utrecht). A qualified independent professional chaired the meeting, with the explicit instruction not to strive for consensus (or dissimilation), but to stick close to the group result as it emerged. The authors had no role in this process. Detailed notes were made, subject to the confirmation of all individual experts. The chairman also monitored the time and assured equal input from all panel members on each topic. With permission of the experts, the panel meeting was recorded with a voice recorder; the recordings were transcribed verbatim, and the unaltered verbatim report was sent to all panel members for confirmation. We allowed for (minimal) changes only for clarification purposes. This amended report was incorporated into the final data synthesis (available on request); it also enabled the panel members to check our summarizing statements.

**Table 3:** Characteristics of panel members.

Panel characteristics		Panel 1 (N = 13)	Panel 2 (N = 68)*
**Gender (female), n (%)**		9 (69.2)	63 (92.6)
**Occupation, n (%)**			
** **	Caregiver**	6 (46.2)	43 (63.1)
** **	Research	7 (53.8)	3 (4.4)
** **	Management	-	5 (7.4)
** **	Other	-	13 (19.1)
**Occupational field**			
	Public health	4 (30.8)	-
	Perinatology	4 (30.8)	-
	Epidemiology	3 (23.1)	-
	Geriatrics	2 (15.3)	-
**Working area**			
** **	National	3 (23.1)	-
** **	High urbanization area	9 (69.2)	54 (79.4)
** **	Other	1 (7.7)	9 (13.2)
Working experience**			
** **	<5 years	-	42 (61.8)
** **	5–10 years	-	6 (8.9)
	>10 years	-	16 (23.5)

* Missing in 5.9–7.9% of cases

** Including student midwives

Panel 2 consisted of attendees of the RCPSW symposium on vulnerability in pregnancy, which took place on November 20^th^, 2014. A total of 68 members were present and agreed to participate. Sociodemographic characteristics are listed in the right column of Table 3.

During the symposium, each panel member was handed an abridged copy of the questionnaire containing tasks 2 through 5. After a general introduction by the research team, and instruction on the schedule of the meeting, each task (2–5) was introduced separately. All panel members filled out responses individually. The response forms were collected and count data were analyzed during the break. At the end of the symposium, the initial results could be presented back to the panel members as a group, for finalization. The feedback provided was incorporated (see below).

#### Tasks not presented in this paper

Tasks 1, 6 and 7 of the pen and paper questionnaire are not presented in this paper. These were completed by panel 1 only, the general research experts, due to time constraints and, in the case of task 1, due to the requirement of in-depth theoretical knowledge or research experience. A short explanation of each task was provided for completeness.

Task 1 invited experts to compare by a Venn diagram technique their definition of vulnerability with the concepts of self-sufficiency, deprivation, frailty and susceptibility. Task 6 contained open-ended questions on ethical aspects of vulnerability in particular on the responsibility for its improvement. Task 7 required judgment on four hypothetical persons/clients. Experts of panel 1 were invited to indicate the degree of vulnerability (not, partially, yes) and to point out the characteristic(s) to be removed or changed first to lower the degree of vulnerability. Task 1 data supported preparation work for the meeting, as the information provided informed the research team of the heterogeneity of terminology among the attendees. Task 6 and 7 data were not pertinent to this paper.

## Results

The duration of panel 1 was about 2 hours, the net duration of panel 2 was about 1 hour. From a procedural perspective the participants of panel 1 provided about equal input. Panel 2 was too large to elicit individual comments of all persons being present at all tasks, but discussion was lively with active involvement of most. Most panel 2 members had previous experience in discussing these topics on RCPSW meetings. The tasks 2, 3, 4 and 5 (original numbering) are presented here for both panels simultaneously.

### Task 2: Elements of vulnerability

The top 11 elements of vulnerability according to panel are listed in Table 4 (columns 1 through 4; column 5 and 6 describe the position of the elements in the model that is presented in subsequent section). Although panels differ in their emphasis on specific elements, this difference seems a matter of terminology: panel 1 primarily uses conceptual terms while panel 2 prefers operational terms (i.e. ‘lack of material resources’ [conceptual] vs. ‘living in a deprived neighborhood’ [operational]). Taking this phenomenon into account, we regarded 4 overarching components as core to vulnerability according to both panels: (1) having insufficient material resources of various kinds; (2), being and feeling unable to take responsibility for one’s health; (3) partaking in unhealthy or risky activities and behaviors; and (4) experiencing inadequate social support.

**Table 4:** Top 11 Elements of vulnerability according to panel (Task 2), and their pathway position in the vulnerability model (Task 4).

Domain	Elements of Vulnerability	Panel 1	Panel 2	Pathway to unhealthy	Pathway to healthy
R	Lack of material resources	1	11	General risks	Natural prognosis, Professional care, Self care
C	Lack of ability to take responsibility for one's health	2	5	Specific risks	Professional care, Self care
R	Low education	3	2	General risks	Professional care, Self care
C	Lack of motivation	4	-	Specific risks	Professional care, Self care
C	Negative perception of situation	5	7*	Specific risks	Natural prognosis, Professional care, Self care
E	Living in a deprived neighborhood	6	1	General risks	Natural prognosis, Professional care
E	High risk occupation	7	4	General risks	Natural prognosis
M	Substance abuse	8	-	Specific risks	Natural prognosis
R	Small social network	9	-	General risks	Self care
SE	Low (preventive) health care accessibility & quality	10	5	Preventive care	Professional care
SE	Lack of insurance coverage	11	7*	Preventive care	Professional care
C	Unhealthy activities and behaviors	-	7*	Specific risks	Natural prognosis, Self care
R	Low income / poverty	-	3	General risks	Natural prognosis
C	Insufficient coping	-	10	Specific risks	Natural prognosis, Self care

*Elements with the equal sum score. R = Resource; C = Coping; E = Environment / exposure / etiological factor; M = Manifestation; SE = Self-efficacy.

### Task 3: Existing definitions of vulnerability

When the definitions were ranked according to their sum score of preference, the results for both panels were nearly identical, with 6 generally preferred definitions. The top 6 definitions of vulnerability are displayed in Table 5. All preferred definitions emphasize the etiological biological pathway from vulnerability to becoming ill, more than the pathway which emphasizes the impaired return to a healthy state, if care and self-care are insufficient due to vulnerability. Panel 1 members commented that no perfect definition of vulnerability exists, but that they preferred an imperfect yet good compromise above having no definition at all or only 'pure' definitions which are partial. Arguments in favor of a compromise were the wish to aid policy makers in decision-making regarding resource distribution, the need to increase clinical acuity among young care professionals for the many ways vulnerability is at work, and the everyday experienced need to harmonize registries and guidelines to create consistency in multidisciplinary treatment or care transfers.

**Table 5:** Top 6 definitions of vulnerability according to panel.

Definition of Vulnerability	Panel 1	Panel 2
Frailty is a dynamic state affecting an individual who experiences losses in one or more domains of human functioning (physical, psychological, social), which is caused by the influence of a range of variables and which increases the risk of adverse outcome.	1	4
Populations at risk for poor physical, psychological, and/or social health.	2	1
Vulnerable populations are groups at increased risk for poor physical, psychological, and social health outcomes and inadequate health care.	3	2
Vulnerability is a multidimensional construct reflecting a convergence of many risk factors at both the individual and community levels, which influence health and healthcare experiences.	4	5
Vulnerability is the susceptibility to harm resulting from the interaction of risk factors and supports and resources available to individuals and group.	5	-
To be vulnerable means to face a significant probability of incurring an identifiable harm while substantially lacking ability and/or means to protect oneself.	6	6
Frailty is an accumulation of deficits across physical, psychological, and social domains.	-	3

The view of panel 1 is represented by 4 statements. First, vulnerability primarily results from the interaction of the person and his/her environment (social, physical). The degree of vulnerability is the net result of risk increasing and risk reducing (i.e. protective) factors in this interaction. Second, this interaction is bidirectional: risk factors may affect the person's resilience, and through the person's inadequacy to respond to challenges, the environmental supports (protective factors) may be depleted, further increasing the strain on the person.

Third, vulnerability is not a simple summation of risk and protective factors, but more so the convergent impact of interacting risk factors. A person may be able to withstand a certain risk or risky situation, but may be unable to continue to do so if another specific stressor is introduced which, due to its interaction with the other risk factors, disturbs the existing delicate equilibrium, resulting in the person becoming susceptible to adverse health outcomes: expert members recalled their experience that in seemingly equal situations of vulnerability and deprivation, some women experience severe effects, while others do not.

Finally, vulnerability does not only influence the person itself, but also his/her significant others, such as the partner, family or informal carer, which is part of the above bidirectionality of the interaction. When explicitly asked to select (mutually blinded) the 2 best definitions out of the 6 generally preferred definitions, a great majority of panel 1 members (71% of votes) selected the same 2 definitions that best matched their views. These are presented here as P1-1 and P1-2 respectively, with their source.

*Definition P1-1: Vulnerability is a multidimensional construct reflecting a convergence of many risk factors at both the individual and community levels, which influence health and healthcare experiences [22]*.

*Definition P1-2: Vulnerability is the susceptibility to harm resulting from the interaction of risk factors and supports and resources available to individuals and groups [23]*.

In the discussion with panel 2 members—who were usually active caregivers in the perinatal field—the increased probability of adverse outcome [after health care interaction] received most attention, more than the nature of reinforcing mechanisms as in panel 1. Consequences prevailed over mechanisms. After the discussion, this panel too was invited to vote for the 2 best definitions. The majority (63%) selected the following:

*Definition P2-1: Frailty [vulnerability] is a dynamic state affecting an individual who experiences losses in one or more domains of human functioning (physical, [psychological, social), which is caused by the influence of a range of variables and which increases the risk of adverse outcome [16]*.

*Definition P2-2: Vulnerable populations are groups at increased risk for poor physical, psychological, and social health outcomes and inadequate health care [24]*.

In the discussion, a tentative definition is presented that combines the strengths of the above definitions and perspectives of both panels, and gives credit to the arguments put forward.

### Task 4: Competing models of vulnerability

**Fig 2:**
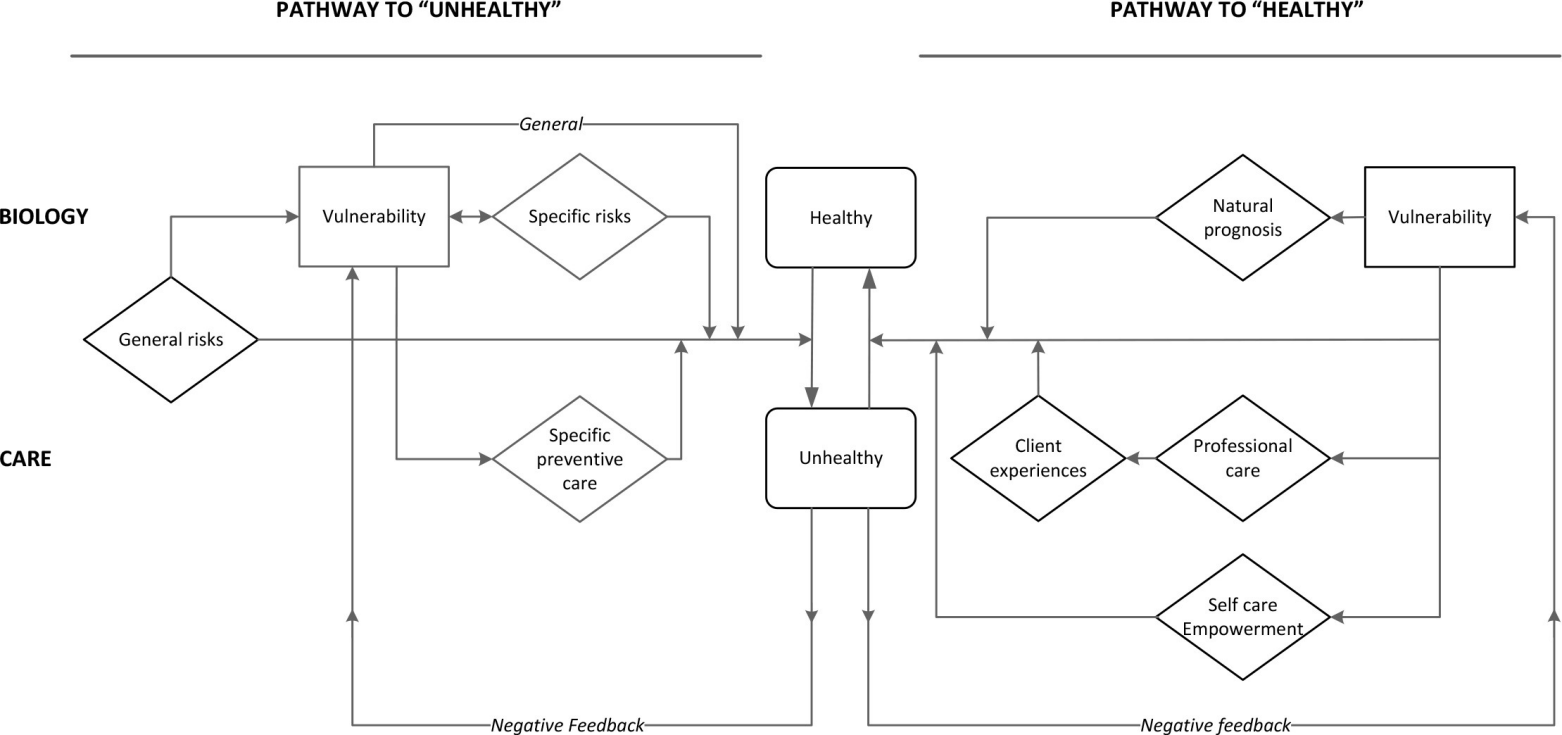
Conceptual model of vulnerability for research and practice.

Both panels had difficulty determining the model that best suited both their preferences on the elements and the assumed relations among all elements. The underlying difficulty appeared to be the reflection of the multitude of pathways into one model or scheme. Consequently, none of the available models was clearly preferred and some experts had circled all or none of the models. During the discussion, however, an agreed overarching scheme/conceptual model (Fig 2) could be defined, based on 3 principles.

The first principle was the acknowledgment of the existence of two separate pathways between vulnerability and health (e.g. adverse outcome, illness): the pathway to adverse outcomes (pathway to becoming unhealthy) and the pathway of recovery, once being unhealthy (pathway to healthy). It was recognized that these pathways are not symmetrical in the sense that the risk leading to ill health not necessarily prevents its recovery, and that preventive factors on the way to an adverse outcome do not necessarily improve an adverse outcome once it occurs. The figure conveniently does not make a distinction between short and long-term factors.

The second principle was the distinction between biological etiological pathways on the one hand and care pathways (either preventive, curative or supportive) on the other hand. Care pathways are determined by the national health system and the public social care structure, much more so than biological factors. In Fig 2, the upper part reflects the direct etiological pathways, the lower part the pathways through care mechanisms.

The third principle (see also the result of task 2) was the presence of reinforcing feedback loops of factors through ill health, which causes vulnerability to create greater and more persistent adverse effects, than might be expected from the factor in isolation. The figure shows the most pertinent negative feedback loops; in data analysis these interactions are reflected in specific forms of epidemiological analysis.

Next, the resulting conceptual model of vulnerability is shown. The term 'pathway' both refers to a specific process, and, in epidemiological analysis, to a probability that some change is induced by the factor involved.

#### Conceptual model of vulnerability

The left side of the model represents the pathway to adverse outcomes. It encompasses general and individual risk factors and the (lack of) uptake of specific preventive care services (e.g. lack of uptake of immunizations by specific populations [3]). General risks, such as gender, age or living in a deprived neighborhood increase vulnerability and the susceptibility to adverse outcome, regardless of their interaction with specific risk factors [21]. Specific risk factors are individual risk factors such as substance use and personal resources, such as social support, social network and coping skills. These specific risk factors reinforce one another and their combined effect influences the degree of vulnerability. Taken together, vulnerability then becomes the net result of general and specific (individual) risk factors and availability of personal resources, regardless of socio-economic strata.

Moving to the right side of the model, the pathway of recovery represents the process of recovery given the degree of vulnerability. This process runs through the natural prognosis and professional and self-care. The natural prognosis is inversely related to vulnerability as the risk factors that contributed to the occurrence of adverse outcomes are generally still in place (e.g. recovery rates of smokers vs. non-smokers differ [25–26]). At the same time, vulnerable individuals are a challenge in professional care as they require complex and often interdisciplinary case management [27] and dropout rates are high [28]. Through associated experiences, vulnerable individuals subsequently develop the tendency to avoid professional care altogether [29].

Finally, self-care relies heavily on health literacy, which in turn is strongly related to general risk factors (i.e. to vulnerability). Estimates of health illiteracy in the general populations vary from 30–50% [30–32].

### Task 5: Diagnosing vulnerability in routine care

If invited to choose one preferred method to assess vulnerability in routine situations or research, the majority of both panels indicated a face-to-face interview as the preferred method, preferably supported with some training. However, during the discussion both panels advocated a combined use a checklist and face-to-face interview as most effective and efficient since the majority of clients or respondents are sufficiently able to fill out a checklist by themselves, and as a method to decrease interview heterogeneity as training is not always a feasible option. The care professional thus could use the results from a general checklist as the input for the face-to-face interview to assess the client’s degree of vulnerability, perhaps tailored to the context. In words of one of the members from panel 1: “The checklist leads to a hypothesis, which is then tested during the interview.” In the case of checklist barriers that cannot be overcome with support, assessment should rely on thorough face-to-face interviewing only.

## Discussion

A Delphi-like procedure with two independent panels of experts clearly showed shared views on the definition of vulnerability and its measurement despite their different backgrounds (general vs. specific). The following shared conceptual definition of vulnerability was agreed upon: *'Vulnerability is a dynamic state that reflects converging effects of a set of interacting and amplifying personal and environmental factors*, *which together increase an individual’s susceptibility to ill health and which hampers the recovery process to normal health once ill health has occurred*.*'* More importantly, the process generated an agreed universal model of vulnerability, which can guide data collection, data analysis, and policy development. The three guiding principles are: pathways to ill health vs. return pathways to health; division between care factors vs. biological and social factors; the mechanism of risk accumulation and reinforcing interaction between person and environment. The agreed model shown in Fig 2 unites seemingly different views of previous authors into one consistent framework. The recognition that vulnerability not only initiates disease but also impacts prognosis, and interferes with recovery, creates opportunities for separate, yet consistent research and care options to decrease vulnerability consequences.

A standard list of measurable components of vulnerability has become available from our study, which can be grouped into four areas: having insufficient material resources of various kinds, being and feeling unable to take responsibility for one’s health, partaking in unhealthy or risky activities and behaviors, and experiencing inadequate social support. Within these four groupings, convergence on the 'best set' was less marked. It appears that within fields like care for the elderly, mental health, or perinatal care, particular vulnerability components have different relevance.

The main difference between panels was a general preference by panel 1 (foremost researchers with various backgrounds) for a definition where the interacting and reinforcing mechanism was explicitly stated, while panel 2 (foremost caregivers) preferred at least full account of the multiplicity of adverse health outcomes (biological, functional and social health states). We explain this difference in emphasis by the difference of professional focus and not by a mismatch between the general concept and a 'perinatal' concept of vulnerability.

### Practical implications

The presented definition and model are designed with translation to multiple disease areas and practical application in mind, such as child services, mental health care, care for the chronically ill, and care for the elderly. The model offers a framework to understand interactions between types of risk and protective factors; the elements associated with each factor will have to be determined separately for each domain through e.g. literature research, consensus amongst experts, and consultation of administrative sources. Also, implementation in the social care domain can be considered, extending the conventional socio-economic vulnerability approach.

However, the implementation of the preferred measurement by checklists and subsequent interviews is not self-evident. At least two barriers may be encountered.

Routine non-research settings such as a social service desk, an ambulatory care unit or a prenatal screening unit will consider the cost of information collection in terms of acquisition time and information technology, versus the direct gains from a 'better' or more consistent measurement of vulnerability. Gains are considered not only for the client but also the organization. To some extent this perception of 'cost' depends on the current awareness of many insufficiencies and inefficiencies in the care for the vulnerable or deprived. If health inequalities are unnoticed, uncommon, or, have low priority, systematic vulnerability measurement will meet resistance. An argument in favor is that at least in perinatal care, the systematic approach ultimately seems time-saving [27], and we observe that increasingly the practical usefulness of checklists for vulnerability is accepted in clinical intake procedures.

Another barrier relates to lack of professional awareness and the feeling of embarrassment which professionals experience when they face a case of vulnerability. Lack of intervention options, presence of communication barriers, and the expectation that adequate care is time consuming, all contribute to the reluctance to give full attention to vulnerability-related care insufficiencies [33–34].

Societal and professional resistance may also be expected with 'too' good a measure of vulnerability. Systematic measurement inevitably detects more cases of vulnerability and more adverse, perhaps preventable, consequences. Better measurement induces questions on responsibilities for the inadequacies observed, which few like to address as no easy solution is present in view of the intersectoral nature of vulnerability, especially in resource-constrained settings. Apart from hesitation to measure adequately, societal resistance is reflected in changing views on the degree to which vulnerability concerns are a personal responsibility, rather than a society’s responsibility to its members. Each member of Panel 1 addressed his/her ethical position towards health inequalities and its sources; this view might affect the model. As was already clear from the choice of elements, consensus was presented on the mechanisms of vulnerability and the pathways to ill-health and recovery. But, while the majority view considered health outcome inequalities as undesirable, the extent to which society and the medical system vs. the individual was responsible for improvement, differed. Generally, the clinical and public health system were responsible once adverse outcomes are manifest; thus, health access inequalities were rejected [5]. Different views existed on responsibilities for the non-medical part of the process. The equal-opportunity-for-all principle for most experts involved active societal efforts to reduce health outcome inequalities also in the social domain. For all, the model was suitable to *reflect* various ethical views on vulnerability, in terms of the choice of the elements, the pathways to be influenced, and the methods of influence.

Despite these caveats, which will require careful attention to information policy, we believe the population and the clients have a right to know what affects their prospects, as indeed this is the only way to involve them in solutions. Health care insurers could do their part by creating arrangements that compensate for the added burden of good care to these clients.

### Strengths and limitations

The strength of this study is the generalizability of our model. The mechanisms described are general and the model incorporates both public health and clinical care elements, all of which are supported by international papers. The model pertains specifically to health and health care in developed countries, as the Dutch health care system is similar to other Western countries. In underdeveloped countries vulnerability is frequently an issue of life and death, thus the magnitude of the effects can be more severe. However, the studies on so called 'responsiveness' of health systems (WHO term [34]), which combining data from many countries with quite diverse stages of development, clearly show that the mechanisms through which vulnerability, education, and welfare act on health are quite universal [6].

Another strength of our study was the variety of backgrounds of experts. While the preparatory work to equalize information level was maximized, this did not necessarily imply that all experts should or would agree. There is a sharp difference between shared concepts, stated in various terms (scenario 1), and different latent concepts, even when terms superficially suggest ideas converge (scenario 2).

Scenario 2 could have occurred; if this had been the case, that result would have been reported. Actually, scenario 1 was the case, and congruence was beyond our expectation in particular across panels. We never required the experts to converge, while this also was unlikely to happen having invited esteemed professionals with a view. In the end, scenario 1 was more applicable: the variety of experts indeed turned into a strength of our study, suggesting the robustness and generalizability or the results. Third, this study made maximum use of available literature from different views within different domains. During the panel meetings, apparently different literature views could be reconciled and together with features of different domains could be incorporated into one general, overarching conceptual model. Fourth, this study explicitly addressed the practical application of the vulnerability concept in routine care (i.e. measurement); all elements put forward by members form both panels can be reliably assessed with a checklist. Even though the use of checklists has its limitations (i.e. socially desirable answers, impaired literacy or other barrier), using checklists is common practice in other fields [35] and has also been advocated within pregnancy care [27, 36]. Finally, this study is repeatable; all study materials are available on request.

A limitation of this study is that there was one, national, generic expert panel, which we contrasted with only one field-specific group (i.e. perinatal care). The extension to more contexts (i.e. fields and countries) is welcomed and could induce further refinements or modifications. Second, the full model and its pathways need to be tested further. To this aim we are currently introducing a vulnerability checklist in a large, prospective cohort of pregnant women in the Netherlands, which aims to disentangle the interaction of risk factors and their convergent impact on adverse outcomes. Finally, filling out a checklist is a cognitively demanding task, which some individuals might not be able to do (due to for instance, a language barrier or intellectual disability) or perceive as troublesome. In the former case, where these barriers cannot be overcome, we recommend vulnerability assessment to rely on a thorough interview with a trained professional only.

### Conclusions

A universal conceptual model of vulnerability, with a proposed measurement approach, has been developed. The concept can be applied in perinatal services and tested further in research and practice in multiple disease areas and subpopulations. Uniformity in definition and concept will benefit researchers, policy makers, care professionals and clients alike. It will facilitate comparability across research results, across subpopulations for specific care, and allow for more equitable resource allocation in care. Admittedly, to disentangle the intricate interactions of reinforcing risk factors and their effect on health and health-related adverse outcomes, and to develop effective and feasible intervention strategies, more research is needed.

## Supporting information

S1 Appendix Vulnerability questionnaire (original language). (PDF)

S2 Appendix Vulnerability questionnaire (English translation). (PDF)

S3 Appendix Literature search ‘Blocks’ for vulnerability used in embase. (PDF)

S1 Table Key papers on vulnerability included in the reader that was sent to Panel 1 members prior to the expert meeting. (PDF)

S2 Table 29 Elements presented for the common vulnerability concept. (PDF)

S3 Table List of 24 Existing vulnerability definitions as included in the written questionnaire. (PDF)

## Abbreviations

RCPSWN: Regional Perinatal Consortium South-West Netherlands
SGA: Small for Gestational Age
R: Resource
C: coping
E: Environment / exposure / etiological factor
M: Manifestation
SE: Self-efficacy
