# Supplementary material for: Towards a universal concept of vulnerability: Broadening the evidence from the elderly to perinatal health using a Delphi approach
Source: PLoS One. 2019 Feb 20;14(2):e0212633. doi: 10.1371/journal.pone.0212633 (PMC6382270; doi:10.1371/journal.pone.0212633)

VRAGENLIJST KWETSBAARHEID

Consensus studie ‘kwetsbaarheid’

11 November 2014

N. de Groot, G.J. Bonsel et al.
n.degroot@eur.nl

Inleiding

Voor u ligt de leidraad voor de consensus-bijeenkomst over het begrip ‘kwetsbaarheid’. Het heeft de opzet van een vragenlijst, maar is vooral bedoeld om systematisch een aantal – in onze ogen – kernvragen te bespreken

Kwetsbaarheid is een breed begrip dat verschillend wordt geïnterpreteerd en in verschillende domeinen wordt toegepast: iemand kan kwetsbaar zijn in relatie tot (het vinden of verrichten van) arbeid of, in ons geval, kwetsbaar zijn in relatie tot gezondheid. Men neemt algemeen aan dat er een wederzijds verband bestaat tussen kwetsbaarheid en gezondheid: kwetsbaarheid draagt bij aan het ontstaan van slechte gezondheid, en een slechte gezondheid draagt bij aan het ontstaan en verergeren van kwetsbaarheid.

De aanleiding voor deze bijeenkomst is dat wij ons als organiserende onderzoekers bezig houden met geboortezorg. Daar speelt kwetsbaarheid een grote rol. Kwetsbare zwangeren is het thema van een grote regionale (Zuidwest Nederland) ZonMw studie, maar ook in andere grote ZonMw studies van collega’s (Amsterdam, Utrecht) speelt dit thema een sleutelrol. De Nederlandse geboortezorg uitkomsten vallen internationaal op, niet alleen vanwege het gemiddeld hoog niveau van ongewenste uitkomsten (o.a. ‘babysterfte’) maar ook door relatief grote verschillen, vooral in grote steden en i.v.m. achterstand. Zorgexperimenten in de geboortezorg zijn erop gericht kwetsbaarheid en de gevolgen daarvan te verminderen.

In zowel zorgexperimenten als gesprekken met gemeenten, GGD, en verzekeraars bleek ‘kwetsbaarheid’ een veel breder thema. Kwetsbaarheid, en daarop gericht beleid van gezondheidszorg, sociale stakeholders, gemeenten en verzekeraars kwamen we tegen bij migranten, Jeugdzorg, zwangeren, pasbevallen moeders en hun baby, ouderen, chronisch zieken, psychiatrisch patiënten, dak- en thuislozen, etc. Zelf zijn wij ook betrokken bij ouderen en psychiatrisch zieken. ‘Kwetsbaarheid’ heeft een sleutelrol bij toekomstige policyvorming voor zorg en welzijn. Deze policy is gericht op decentralisatie van allerlei zorg en welzijnstaken naar de gemeenten, die primair geacht worden in ieder geval zorg aan de meest kwetsbaren te leveren; minder of niet aan de zelfredzamen. Recent stelden gemeenten aan ons de vraag het kwetsbaarheids-begrip van de geboortezorg in te passen / aan te passen aan het begrip ‘zelfredzaamheid’, zoals dat sinds kort wordt toegepast. Ook ZonMw verwacht over en weer van onderzoekers dat ze kernbegrippen afstemmen (als mogelijk): zo lijkt het aantrekkelijk samenhang te zoeken met onderzoeken naar monitoring van kwetsbare groepen door gemeenten zoals onderzocht wordt in Brabant.

Hoewel de inspiratie gemeenschappelijk is, blijkt het zowel een wetenschappelijke als maatschappelijke uitdaging om tot een voldoende eenduidige definitie van het begrip kwetsbaarheid te komen.
Binnen het wetenschappelijk perspectief op kwetsbaarheid staat de etiologische rol van kwetsbaarheid centraal: wat is kwetsbaarheid, wat zijn determinanten van kwetsbaarheid, wat zijn de gevolgen voor gezondheid en – volgens sommigen – gezondheidszorg gebruik, en is kwetsbaarheid te meten? Is

kwetsbaarheid bv. een kenmerk van het individu of juist van de groep (persoonlijk of sociaal / omgeving)? Ook is er de vraag welk aangrijpingspunt voor interventie moet worden gekozen: de kwetsbaarheid zelf, of de gevolgen van kwetsbaarheid, met het achterliggende idee dat alleen gevolgen bestrijden mensen niet weerbaarder en zelfredzamer maakt.
Vanuit een maatschappelijk perspectief is van belang tot waar het verbeteren van kwetsbaarheid of de gevolgen daarvan de eigen verantwoordelijkheid is van de autonome zelfstandige burger en waar de verantwoordelijkheid van de overheid / gemeente begint. Dat is de definitie van een grens die deels politiek / ideologisch bepaald is (en volgens critici ook financieel). De individuele verantwoordelijkheid omvat zelfzorg, zelfredzaamheid en zelforganisatie, maar wij vermoeden dat de grens tussen eigen verantwoordelijkheid en de verantwoordelijkheid van de maatschappij (tot en met zorg achter de voordeur) niet door iedereen gelijk wordt ingevuld, ook niet door onderzoekers. Die grens bepaalt evenwel wel waarin moet worden geïnvesteerd om (de gevolgen van) kwetsbaarheid terug te dringen: een verplichte verzekering voor minima met geen of een beperkt eigen risico, onderwijs en gezondheids- en opvoedingsvoorlichting gericht op burgers in een achterstandssituatie, of uitbreiding / meer toegankelijk maken van het werkaanbod voor laaggeschoolden. En voor onderzoekers bepaalt die grens variabelen die worden geïncludeerd / geëxcludeerd in het onderzoeksontwerp en vaak ook een deel van het analysemodel. Ook is die grens vaak erg belangrijk als ‘praktische’ drempel om zorg of bemoeienis te starten (zie de zelfredzaamheidsmatrix).

Wij gaan in deze studie uit van een breed toepassingsperspectief van kwetsbaarheid, dat wil zeggen, het begrip is gezondheidsgerelateerd en mede toepasbaar voor ouderen, psychiatrisch patiënten, kinderen, chronisch zieken etc. De nadruk op gezondheid leidt ertoe dat er altijd relaties zijn met publieke en individuele gezondheidszorg.

Er zijn meerdere wetenschappelijke modellen van kwetsbaarheid in deze context en daarvan afgeleide tools om kwetsbaarheid vast te stellen. De praktijk vraagt enige convergentie (zie hiervoor). In het kader van *‘science meets practice’* willen wij met u onderzoeken of een meer eenduidige, algemeen bruikbare definitie van kwetsbaarheid mogelijk is (en zo nee, waar dat op vastloopt). Dit document heeft 7 onderdelen. De onderdelen 1 t/m 5 richten zich meer op *‘science’*, onderdelen 6 en 7 richten zich meer op *‘practice’*. Elk onderdeel heeft een leidende vraag. Geeft u kort (evt. in steno) antwoord in de tekstvakken. De onderdelen zijn:

1) Overeenkomsten en verschillen tussen wetenschappelijke kwetsbaarheidsbegrippen
2) Plaats(en) kwetsbaarheid in een oorzaken-keten van gezondheid
3) Elementen van kwetsbaarheid
4) Ethische aspecten van kwetsbaarheid
5) Bestaande definities uit gepubliceerde wetenschappelijke artikelen
6) Kwetsbaarheid: hoe stellen we dat vast?
7) Casuïstiek: concrete gevallen: kwetsbaar of niet?

De bijeenkomst op 11 november wordt gericht voorbereid a.d.h.v. uw antwoorden.
Wilt u ons daarom uiterlijk 31 oktober de vragenlijst retour sturen.

Onderdeel 1: Overeenkomsten en verschillen tussen wetenschappelijke kwetsbaarheidsbegrippen

In de kwetsbaarheidsliteratuur worden verschillende begrippen gebruikt om (een vorm van) kwetsbaarheid aan te duiden. Deze begrippen kwamen wij het meeste tegen (I t/m V):

I. Kwetsbaarheid (vulnerability)
II. Zelfredzaamheid (self-sufficiency)
III. Achterstand (deprivation)
IV. Fysieke kwetsbaarheid (frailty)
V. Specifieke kwetsbaarheid (susceptibility)

Met opzet hebben wij zowel de Nederlandse als de Engelse vertaling gegeven. Deze begrippen zijn deels verschillend maar vertonen ook overlap. In dit onderdeel van de vragenlijst proberen we hier meer duidelijkheid in te krijgen. Eerst volgen twee algemene vragen. Daarna zullen de begrippen op drie punten worden vergeleken t.o.v. begrip I. Kwetsbaarheid (vulnerability).

Vraag 1: Zijn er volgens u nog andere (belangrijke) begrippen die niet in de lijst staan? Zo ja, kunt u aangeven welke? Geef het liefst zowel de Nederlandse als Engelse aanduiding.

VI. …..
VII. …..
VIII. …..

Vraag 2: Geef voor elk begrip aan binnen welke context of contexten het begrip op dit moment volgens u een herkenbare rol speelt.

	GGZ	Ouderen	Zwangeren	Jeugdzorg	Chronisch zieken
I. Kwetsbaarheid (vulnerability)					
II. Zelfredzaamheid (self-sufficiency)					
III. Achterstand (deprivation)					
IV. Fysieke kwetsbaarheid (frailty)					
V. Specifieke kwetsbaarheid (susceptibility)					
VI. …..					
VII. …..					
VIII. …..					

Vanaf nu volgt de vergelijking met I. Kwetsbaarheid (vulnerability) op de volgende drie punten:

A) Continuüm: waar staat het begrippenpaar op het continuüm ‘individueel – collectief/samenleving’.
B) Reikwijdte: in hoeverre is het begrip smal of juist veelomvattend bij vergelijking met het algemene begrip kwetsbaarheid.
C) Specificiteit: waarin is het begrip onderscheidend (ook in meetbare zin)?

Vraag 3: Karakteriseer de begrippen op deze drie punten. Wij beginnen met een uitwerking aan de hand van een voorbeeld, waarbij Genetische kwetsbaarheid (genetic vulnerability) wordt vergeleken met (uw beeld van) begrip I. Kwetsbaarheid (vulnerability).

[BEGIN VOORBEELD]

A) Continuüm

Geef Genetisch kwetsbaarheid (genetic vulnerability) een plaats op dit continuüm door een X te plaatsen waar u vindt dat het begrip Genetische kwetsbaarheid (genetic vulnerability) hoort relatief ten opzichte van begrip I. Kwetsbaarheid (vulnerability).

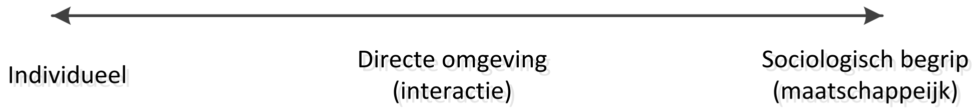

B) Reikwijdte

De paarsgewijze vergelijking wordt gedaan met een Venn-diagram. In een Venn-diagram wordt de verhouding van het begrippenpaar visueel afgebeeld. Hieronder ziet u 5 mogelijke verhoudingen van het begrip Genetische kwetsbaarheid (gKWB) en I. Kwetsbaarheid (KWB):

1. gKWB is geheel onderdeel van I. KWB
2. gKWB is een belangrijk aspect en heeft overlap met I. KWB
3. gKWB is een minder belangrijk aspect en heeft overlap met I. KWB
4. gKWB is staat helemaal los van I. KWB.
5. gKWB is een minder belangrijk aspect en staat helemaal los van I. KWB

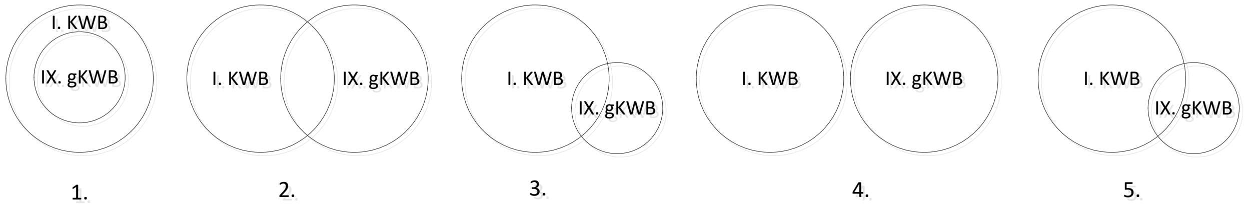

Omcirkel het nummer van het Venn-diagram dat uw mening het best weergeeft. In het voorbeeld hebben wij mogelijkheid 1 omcirkeld.

C) Specificiteit
Geef naar aanleiding van het Venn-diagram wat u heeft getekend aan wat volgens u het onderscheidende is in Genetische kwetsbaarheid (genetic vulnerability) t.o.v. begrip I. Kwetsbaarheid (vulnerability).

Voorbeeldantwoord: gKWB is geheel onderdeel van KWB, omdat genen een oorzakelijke factor hebben in het ontstaan van slechte gezondheid.

[EINDE VOORBEELD]

Hierna beginnen de vergelijkingen van de begrippen II t/m V t.o.v. begrip I. Kwetsbaarheid (vulnerability).

Begrip II. Zelfredzaamheid (self-sufficiency)

Zet bij een kruis bij A), omcirkel een getal bij B) en geef enkele woorden bij C).

A) Continuüm

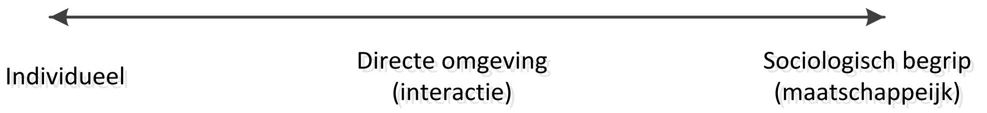

B) Reikwijdte

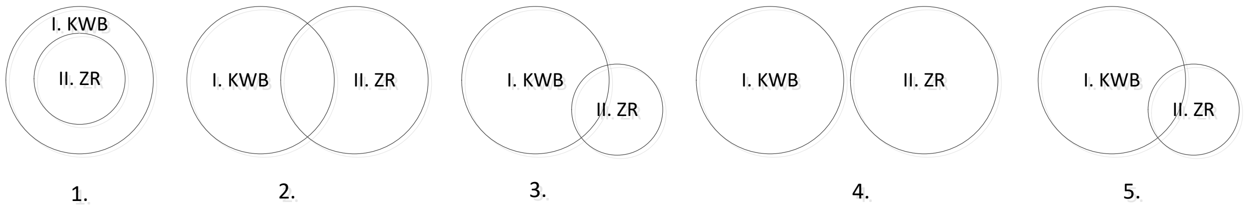

C) Specificiteit: onderscheid tussen begrip II. Zelfredzaamheid (self-sufficiency) t.o.v. begrip I. Kwetsbaarheid (vulnerability).

Antwoord:

Zet bij begrippen III en IV weer een kruis bij A), omcirkel een getal bij B) en geef enkele woorden bij C).

Begrip III. Achterstand (deprivation)

A) Continuüm

Individueel | Directe omgeving (interactie) | Sociologisch begrip (maatschappeijk)

B) Reikwijdte

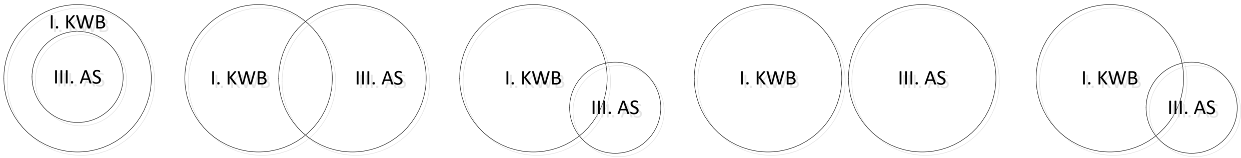

1. 2. 3. 4. 5.

C) Specificiteit: onderscheid tussen begrip III. Achterstand (deprivation) t.o.v. begrip I. Kwetsbaarheid (vulnerability).

Antwoord:

Begrip IV. Fysieke kwetsbaarheid (frailty)

A) Continuüm

Individueel | Directe omgeving (interactie) | Sociologisch begrip (maatschappeijk)

B) Reikwijdte

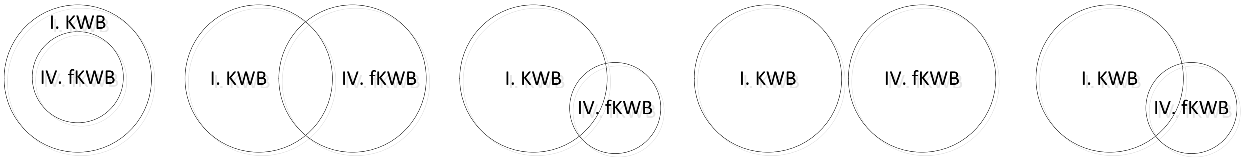

1. 2. 3. 4. 5.

C) Specificiteit: onderscheid tussen begrip IV. Fysieke kwetsbaarheid (frailty) t.o.v. begrip I. Kwetsbaarheid (vulnerability).

Antwoord:

Zet bij begrip V weer een kruis bij A), omcirkel een getal bij B) en geef enkele woorden bij C).

Begrip V. Specifieke kwetsbaarheid (susceptibility)

A) Continuüm

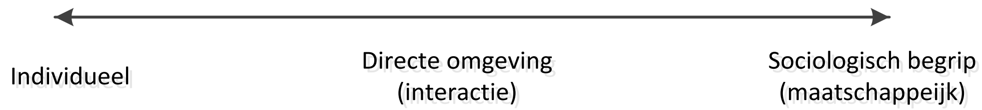

B) Reikwijdte

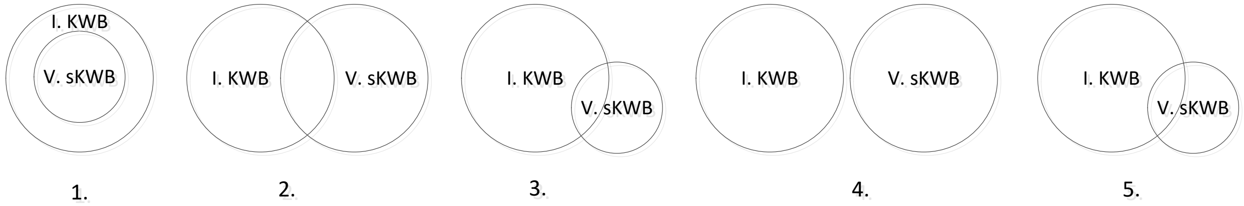

C) Specificiteit: onderscheid tussen begrip V. Specifieke kwetsbaarheid (frailty) t.o.v. begrip I. Kwetsbaarheid (vulnerability).

Antwoord:

Onderdeel 2: Plaats(en) kwetsbaarheid in een oorzaken-keten van gezondheid

Een specifieke definitie van kwetsbaarheid heeft gevolgen voor de plaats van kwetsbaarheid in de oorzaken-keten naar (on)gezondheid: wordt kwetsbaarheid gedefinieerd als aanleg voor het ontstaan van een ziekte of speelt het later (ook) een rol? De positie in de oorzaken-keten is van belang bij de keuze van interventies.
Hieronder ziet u 5 modellen die wij in de literatuur gevonden hebben waarin een oorzakelijke relatie tot (on)gezondheid wordt weergegeven. De afkortingen zijn 'KWB = Kwetsbaarheid', 'D_1 D_n = Determinanten 1 t/m n' en 'G = Gezondheid'. Wij hebben geen voorkeur.

Vraag 4: Ga uit van uw visie op kwetsbaarheid. Kruis de modellen door die u minder / niet relevant vindt voor het begrip kwetsbaarheid. Omcirkel het model dat volgens u in de praktijk het sterkste is.

Model 1

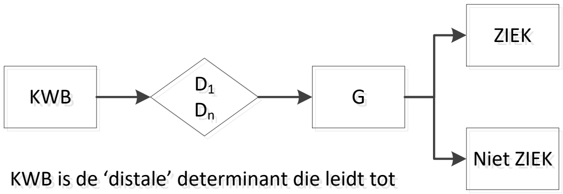

KWB is de 'distale' determinant die leidt tot grotere kans op ongunstige determinanten voor (on)gezondheid.

Model 2

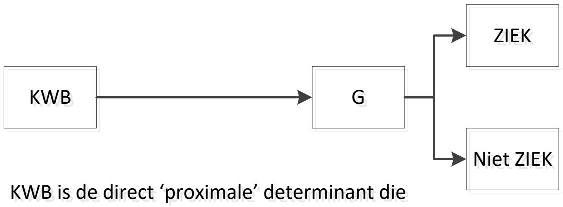

KWB is de direct 'proximale' determinant die leidt tot grotere kans op (on)gezondheid.

Model 3

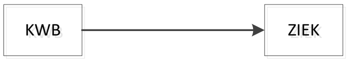

KWB is de direct 'proximale' determinant die bijdraagt aan een lagere kans op herstel. Het gaat hier om het natuurlijke prognostische verloop.

Model 4

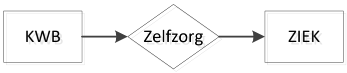

KWB draagt als 'distale' factor bij aan lagere kans op herstel via inadequate zelfzorg.

Model 5

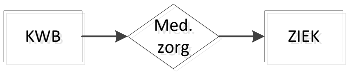

KWB draagt als 'distale' factor bij aan lagere kans op herstel via inadequate verzekerde, medische, zorg

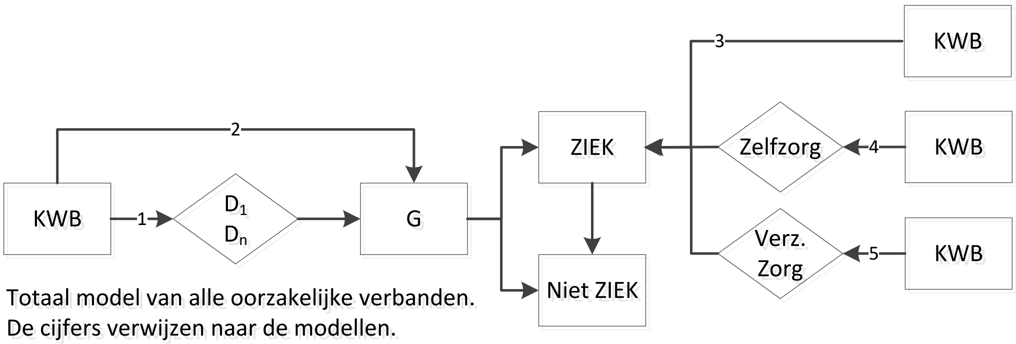

Totaal model van alle oorzakelijke verbanden.
De cijfers verwijzen naar de modellen.

Onderdeel 3: Elementen van kwetsbaarheid

Kwetsbaarheid is een abstract begrip. Zodra we gaan meten wordt duidelijk wat een onderzoeker of instantie onder kwetsbaarheid verstaat en wat niet (meer). Wij noemen dit de elementen van kwetsbaarheid.

Vraag 5: In de tabel hieronder vindt u een lijst met elementen uit de (Engelstalige) literatuur. Ze kunnen overlappen, dat is onvermijdelijk. Geef voor elk element aan of u vindt of dit een element van kwetsbaarheid is. Zijn er volgens u nog andere (belangrijke) elementen die niet in de lijst staan? Zo ja, vul de lijst aan. Op de bijeenkomst wordt de beïnvloedbaarheid van de meest genoemde elementen besproken.

	Elementen	Wel	Deels	Niet
1	Age (low and high)			
2	Female gender			
3	High exposure to risks			
4	High risk occupation			
5	Insufficient coping			
6	Lack of ability to take responsibility for one's health			
7	Lack of insurance coverage			
8	Lack of material resources			
9	Lack of motivation			
10	Lack of reserve capacity			
11	Lack of resilience			
12	Living in a deprived neighborhood			
13	Low (preventive) health care accessibility and quality			
14	Low education			
15	Low income / poverty			
16	Low sense of control and mastery			
17	Low social status			
18	Low social support			
19	Minority group / race / ethnic background			
20	Negative perception of situation			
21	Poor physical health			
22	Poor psychological health			
23	Psychosocial stress			
24	Religion			
25	Small social network			
26	Stigma			
27	Substance abuse			
28	Unhealthy activities and behaviors			
29	…			
30	…			

Gevolgen van kwetsbaarheid

Kwetsbaarheid heeft meerdere gevolgen. Wat staat bij kwetsbaarheid voorop? Wat is de parameter waar het kwetsbaarheid beleid en het succes bij kwetsbaarheid interventie op af word gerekend?

Vraag 6: Omcirkel het nummer van de optie die u het beste vind passen. De meest gekozen optie zal op de bijeenkomst worden besproken.

1. Subjectief well-being begrip
2. Generieke gezondheid
3. Het ontstaan (of uitblijven) van ziekte, m.n. chronische ziekte, handicap of event (bv. ongeval).
4. Mortaliteit (of overleving; verwachte overleving)

Positief en negatief

De voorgaande elementen zijn consequent negatief geformuleerd. Dat is opzettelijk.
De meeste begrippen zijn bipolair, b.v. lage vs. hoge opleiding. Niet altijd is gezegd dat (in dit voorbeeld) hoge opleiding beschermend is, wanneer lage opleiding risicovol is.
Sommige begrippen zijn unipolair: er is niet echt een tegengestelde van.

Vraag 7: Zijn er elementen van kwetsbaarheid die u opgenomen wilt zien die specifiek positief van effect zijn; dus omgekeerd werken aan kwetsbaarheid. We bedoelen dus niet ‘goede’ coping i.p.v. ‘slechte’ coping maar intrinsiek positief werkende elementen, b.v. ‘gelukkig huwelijk / partnerschap’.

1. .….
2. .….
3. .….
4. .….
5. .….

Onderdeel 4: Ethische aspecten van kwetsbaarheid

In dit onderdeel gaat het over de door kwetsbaarheid veroorzaakte ongelijkheid (inequality) in het ontstaan van ziekten en hun beloop. Verschillen in gezondheidsuitkomsten hangen b.v. sterk samen met etniciteit en socio-economische status (SES), twee onbetwiste elementen van kwetsbaarheid. Ook al is dit effect op ongelijkheid deels onvermijdbaar, toch wordt deze ongelijkheid maatschappelijk als zeer ongewenst beschouwd. Hierover hebben we twee vragen:

Vraag 8: Vindt u dat er een ethisch-moreel aspect zit aan kwetsbaarheid? B.v. het individu dan wel de maatschappij moet werken aan het verminderen van (de gevolgen van) kwetsbaarheid? Is dat (evt.) voor u afhankelijk van de beïnvloedbaarheid van kwetsbaarheid? Is aan (de gevolgen van) kwetsbaarheid werken ook een (zorg)taak als de succeskans klein is?

Antwoord:

Vraag 9: Vindt u dat in professionele richtlijnen specifiek aandacht aan kwetsbaarheid moet worden besteed, in die zin dat een professional zich meer moet inspannen om effectief de (gevolgen van) kwetsbaarheid te verminderen of tot nul te brengen?

Antwoord:

Onderdeel 5 : **Bestaande definities uit gepubliceerde wetenschappelijke artikelen**

In de literatuur zijn verschillende definities van kwetsbaarheid te vinden. Een aantal daarvan vindt u terug in de syllabus. Op de volgende pagina vindt u een lijst met 24 definities van kwetsbaarheid.

Vraag 10: Geef de acht definities die u het best vindt passen bij kwetsbaarheid een 2, de acht definities die u minder goed vindt passen een 1 en de acht definities die u het minst vindt passen een 0. Natuurlijk kunt u iets schuiven in de verhouding.

De 10 meest genoemde definities worden op de bijeenkomst verder besproken.

Schrijf a.u.b. een 0, 1 of 2 achter iedere stelling

	**Definitie kwetsbaarheid**	**Oordeel 0 / 1 / 2**
1	Frailty is a dynamic state affecting an individual who experiences losses in one or more domains of human functioning (physical, psychological, social), which is caused by the influence of a range of variables and which increases the risk of adverse outcomes (Gobbens, 2010).	
2	Vulnerable populations are populations at risk for poor physical, psychological, and/or social health (Aday, 1994).	
3	Vulnerability is the propensity of social or ecological systems to suffer harm from external stresses and perturbations (Kasperson et al, 1995 in deFur et al, 2007).	
4	Vulnerability is a multidimensional construct reflecting a convergence of many risk factors at both the individual and community levels, which influence health and healthcare experiences (Shi et al, 2005 in Shi et al, 2008).	
5	Vulnerable groups are social groups who have an increased relative risk or susceptibility to adverse health outcomes (Flaskerud, 1998 in Flaskerud et al, 1998).	
6	Vulnerability is defined as the constellation of past, present and future risk, perceived or real, as a result of the common human experience of risk, the increased vulnerability of the adolescent period, consequences of family disruption, and increased risks of life on the street (Dorsen, 2010).	
7	Vulnerability is the susceptibility to harm resulting from the interaction of risk factors and supports and resources available to individuals and groups (Mechanic et al, 2007).	
8	Vulnerability is an increased susceptibility to health and health care disparities due to a combination of individual and environmental factors (Grabovschi et al, 2013).	
9	Frailty is an accumulation of deficits across physical, psychological, and social domains (Salem et al, 2014).	
10	Vulnerability is a condition of heightened fragility of a population or specific group, and a process that is potentially reversible or avoidable through appropriate interventions (Zarowsky et al, 2013).	
11	Vulnerability is the progressive loss of wellbeing, i.e. health, related to social and economic deprivation (WHO definition in Allotey et al, 2012 in Zarowsky et al, 2013).	
12	Vulnerable populations are groups that are clinically at risk and/or socially disadvantaged (Lewis et al, 2012).	
13	Vulnerability is the universally present relative risk of potential or actual harm from external judgments of endangerment, functional capacity, and socially sanctioned need for intervention (Demi et al, 1995 in Spiers, 2000).	
14	Vulnerability is the experience of exposure to harm which challenge one's integrity (Spiers, 2000).	
15	Vulnerable groups are social groups who experience limited resources and consequent high relative risk for morbidity and premature mortality (London, 2007 in Amin et al, 2011).	
16	Social vulnerability is a precarious economic situation justifying the allocation of welfare benefits and/or resulting in inadequate health coverage (Marmot, 2005 and Ridde, 2007 in Pascal et al, 2009).	
17	To be vulnerable means to face a significant probability of incurring an identifiable harm while substantially lacking ability and/or means to protect oneself (Schroeder et al, 2009).	
18	To be vulnerable means to be substantially incapable of protecting one's own interests (Council for International Organizations of Medical Science [CIOMS] in Schroeder et al, 2009).	
19	Vulnerable populations are groups at increased risk for poor physical, psychological, and social health outcomes and inadequate health care (Aday, 2001 and Flaskerud et al, 1998 in Pitkin Derose et al, 2007).	
20	Vulnerable populations are those at greater risk for poor health status and health care access (Shi et al, 2004).	
21	Vulnerability speaks to susceptibility to health problems, harm, or neglect (Phillips, 1992 en Rogers, 1997 in Bragg Leight, 2003).	
22	Vulnerable populations are populations in which complex medical needs are exacerbated by social needs (Vanderbilt et al, 2013).	
23	Vulnerable people are those who are less likely than average to obtain medical care of an appropriate quality and quantity (Pauly et al, 2007).	
24	Vulnerable populations are groups whose demographic, geographic, or economic characteristics impede or prevent their access to health care services (Blumentahal et al, 1995).	

Onderdeel 6: Kwetsbaarheid: hoe stellen we dat vast?

In dit onderdeel staat het vaststellen van kwetsbaarheid centraal. Het vaststellen van kwetsbaarheid kan betrekking hebben op een regio (Zuidwest Nederland) of op een individueel geval (iemand die bij de gynaecoloog op intake komt). In dit onderdeel gaat het over het individuele geval.
Kwetsbaarheid kan worden vastgesteld door middel van een self-report vragenlijst, in een interview met een zorgverlener of door vaststelling achteraf (registratie).

Vraag 11: Hoe kan binnen de volgende contexten op individueel niveau kwetsbaarheid het beste worden vastgesteld?

	GGZ	Ouderen	Zwangeren	Jeugdzorg	Chronisch zieken
Vragenlijst / checklist professional support					
Vragenlijst / checklist self-report					
Face to face tijdens consult					
Registratie (bv medische gegevens)					

Vraag 12: Geef een voorbeeld van een 'best practice' voor het op individueel niveau vaststellen van kwetsbaarheid.

Antwoord:

Onderdeel 7: Casuïstiek: concrete gevallen: kwetsbaar of niet?

Dit onderdeel bevat 4 casussen.

Vraag 13: Geef aan of u deze persoon kwetsbaar vindt of niet. Als u deze persoon als kwetsbaar beschouwd, geef dan ook aan welk(e) kenmerk(en) weg zouden moeten worden gehaald zodat u deze persoon niet langer als kwetsbaar beschouwd.

Casus 1

Man, 35 jaar, hoog opgeleid, gescheiden, weinig vrienden, voor 80% afgekeurd i.v.m. ernstige darmziekte (colitis ulcerosa), wonende in een gemiddelde wijk, normaal contact met buren.

A) Deze persoon is **wel / deels / niet** kwetsbaar.
B) Omcirkel de kenmerk(en) die zouden moeten worden weggehaald om ervoor te zorgen dat deze persoon niet meer kwetsbaar is.

Man	Weinig vrienden
35 jaar	80% afgekeurd i.v.m. ernstige darmziekte
Hoog opgeleid	Wonende in een gemiddelde wijk
Gescheiden	Normaal contact met buren

Casus 2

Vrouw, 26 jaar, voor de 2^{e} keer zwanger, laag opgeleid, vaste baan, wonend in een achterstandswijk, samenwonend, moeizame relatie, beiden roken, ouders partner bieden effectief hulp in de opvoeding van het 1^{e} kind en het huishouden.

A) Deze persoon is **wel / deels / niet** kwetsbaar.
B) Omcirkel de kenmerk(en) die zouden moeten worden weggehaald om ervoor te zorgen dat deze persoon niet meer kwetsbaar is.

Vrouw	Wonend in een achterstandswijk
26 jaar	Samenwonend
Voor de 2^{e} keer zwanger	Moeizame relatie
Laag opgeleid	Beiden roken
Vaste baan	Effectieve hulp ouders partner

Casus 3

Man, 84 jaar, Marokkaanse afkomst, laag opgeleid, goed gezond, weduwnaar, sterk gelovig, was voorganger in de moskee, spreekt matig Nederlands, volwassen kinderen wonen in Marokko, woont boven een Islamitische slagerij.

A) Deze persoon is **wel / deels / niet** kwetsbaar.
B) Omcirkel de kenmerk(en) die zouden moeten worden weggehaald om ervoor te zorgen dat deze persoon niet meer kwetsbaar is.

Man	Sterk gelovig
84 jaar	Was voorganger in de moskee
Marokkaanse afkomst	Spreekt matig Nederlands
Laag opgeleid	Volwassen kinderen wonen in Marokko
Goed gezond	Woont boven een Islamitische slagerij
Weduwnaar	

Casus 5
Vrouw, 42, van Aziatische afkomst, getrouwd, ongewenst voor de 1^{e} keer zwanger, PhD in theoretische natuurkunde, 60-urige werkweek, regelmatig gebruik alcohol, Aziatische partner spreekt slecht Nederlands, partner heeft actieve kinderwens, woont in en wijk met veel landgenoten, weinig contact in de buurt.

A) Deze persoon is **wel / deels / niet** kwetsbaar.
B) Omcirkel de kenmerk(en) die zouden moeten worden weggehaald om ervoor te zorgen dat deze persoon niet meer kwetsbaar is.

Vrouw	60-urige werkweek
42 jaar	Regelmatig gebruik alcohol
Van Aziatische afkomst	Aziatische partner spreekt slecht Nederlands
Getrouwd	Partner heeft actieve kinderwens
Ongewenst voor de 1^{e} keer zwanger	Woont in een in wijk met veel landgenoten
PhD in theoretische natuurkunde	Weinig contact in de buurt

U heeft nu het einde van de vragenlijst bereikt.
Dank u wel voor het invullen.

Stuur de vragenlijst voor 31 oktober 2014 terug in de bijgevoegde retourenveloppe.

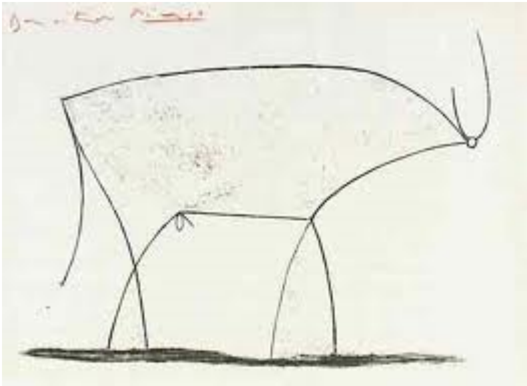

Supplement: S1 Appendix — (PDF) [file pone.0212633.s001.pdf]
