# Supplementary material for: Towards a universal concept of vulnerability: Broadening the evidence from the elderly to perinatal health using a Delphi approach
Source: PLoS One. 2019 Feb 20;14(2):e0212633. doi: 10.1371/journal.pone.0212633 (PMC6382270; doi:10.1371/journal.pone.0212633)

VULNERABILITY QUESTIONNAIRE

Consensus study ‘Vulnerability’

11 November 2014

N. de Groot, G.J. Bonsel et al.

n.degroot@eur.nl

Task 2: **Vulnerability in the cause-effect chain of (un)health**

A specific definition of vulnerability implies consequences for its place in the cause-effect chain of (un)health: is vulnerability defined as a predisposition to developing an illness or does vulnerability (also) play a role at a later stage? The position in the cause- effect chain is important in choosing interventions.

Below you see 5 models that were found in the literature in which the causal relationship to (un)health is displayed. The abbreviations are ‘KWB = Vulnerability’, ‘D_1 D_n = Determinants 1 through n’, ‘G = Health’, ‘ZIEK = Unhealthy’, ‘Niet ZIEK = Healthy’, ‘Zelfzorg = Self care’, ‘Verz. Zorg = Professional care’. We have no preference.

Question 4: Based on your own vision on vulnerability, cross out the models that you feel are less or not relevant to the concept of vulnerability. Circle the model that fits most with the clinical aspect of vulnerability.

Model 1 (Dutch version)

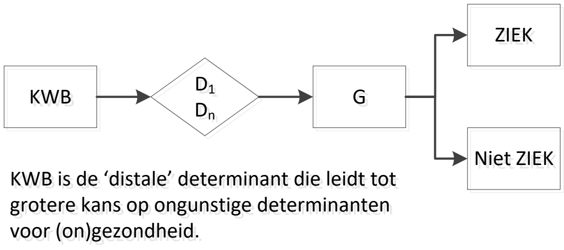

Model 2 (Dutch version)

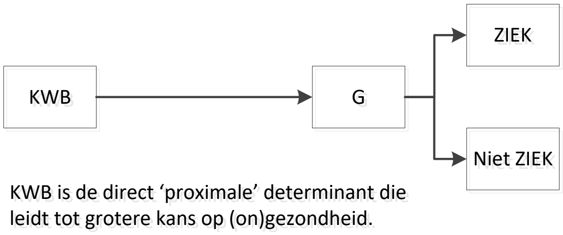

Translation

The abbreviations are ‘KWB = Vulnerability’, ‘D_1 D_n = Determinants 1 through n’, ‘G = Health’, ‘ZIEK = Unhealthy’, ‘Niet ZIEK = Healthy’.

Text below figures

Model 1: Vulnerability is a ‘distal’ determinant that increases the odds of adverse determinants for (un)health.

Model 2: Vulnerability is the direct ‘proximal’ determinant that increases the odds of (un)health.

Model 3 (Dutch version)

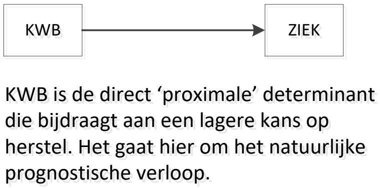

Model 4 (Dutch version)

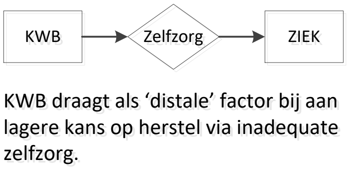

Model 5 (Dutch version)

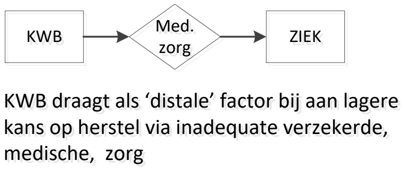

Translation

The abbreviations are ‘KWB = Vulnerability’, ‘D_1 D_n = Determinants 1 through n’, ‘G = Health’, ‘ZIEK = Unhealthy’, ‘Niet ZIEK = Healthy’, ‘Zelfzorg = Self care’, ‘Verz. Zorg = Professional care’.

Text below figures

Model 3: Vulnerability is the direct ‘proximal’ determinant that contributes to lower odds of recovery. This cause-effect chain concerns the natural prognosis.

Model 4: Vulnerability contributes as ‘distal’ factor to lower odds of recovery through inadequate self care.

Model 5: Vulnerability contributes as ‘distal’ factor to lower odds of recovery through inadequate professional care.

Model 6 (Dutch version)

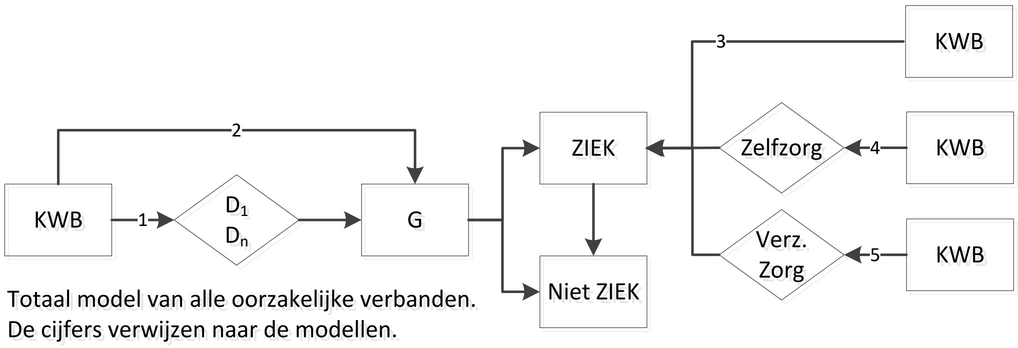

Text below figure

Model 6: Total model of all cause-effect chains. The numbers correspond to the models.

Task 3: Elements of Vulnerability

Vulnerability is an abstract concept. As soon as a researcher or care professional is asked to 'measure' or 'determine' vulnerability status, the answer shows what terms he or she associates with vulnerability and what elements are less or not important. We call the selected terms the elements of vulnerability.

Question 5: The table below contains a list of terms/elements taken from (English) literature. They may overlap, that is unavoidable. Please indicate for each element whether you think this is an element of vulnerability (NO ranking). If you find there are (important) elements missing, please write them at the end of the list. During the meeting the way in which the highest ranked element affect vulnerability is discussed.

	**Elements**	**Important ?**	**Yes**	**Partially**	**No**
1	Age (low and high)				
2	Female gender				
3	High exposure to risks				
4	High risk occupation				
5	Insufficient coping				
6	Lack of ability to take responsibility for one's health				
7	Lack of insurance coverage				
8	Lack of material resources				
9	Lack of motivation				
10	Lack of reserve capacity				
11	Lack of resilience				
12	Living in a deprived neighborhood				
13	Low (preventive) health care accessibility and quality				
14	Low education				
15	Low income / poverty				
16	Low sense of control and mastery				
17	Low social status				
18	Low social support				
19	Minority group / race / ethnic background				
20	Negative perception of situation				
21	Poor physical health				
22	Poor psychological health				
23	Psychosocial stress				
24	Religion				
25	Small social network				
26	Stigma				
27	Substance abuse				
28	Unhealthy activities and behaviors				
29	…				
30	…				

Consequences of Vulnerability
Vulnerability may have multiple consequences. But what consequences are the most prominent? What is the parameter by which the 'vulnerability policy' and success of interventions targeting vulnerability should be (at least) evaluated?

Question 6: Circle the number of the option that you find most fitting. The option that is selected most often will be discussed during the meeting.

1. Subjective well-being
2. Generic health
3. Occurrence (or absence) of disease, specifically chronic conditions, handicap, or event (e.g. accident).
4. Mortality (or survival; expected survival)

Positive and negative
The previously mentioned elements are commonly negatively formulated. This is on purpose.
Most concepts are bipolar (e.g. low vs. high education). Not always is high education (in this example) a protective factor, if low education is a proven adverse risk factor. Some concepts are unipolar: they lack an opposite.

Question 7: Are there elements of vulnerability that you would like to see added to the concept that are specifically positive of effect, thus the opposite of vulnerability? We do not refer to 'good coping' instead of 'bad coping', but intrinsically positive elements, such as 'happy marriage or partnership'.

1. .….
2. .….
3. .….
4. .….
5. .….

Task 5: Existing definitions from published scientific articles

Scientific literature reports several definitions of vulnerability. A few of those you can find in the reader accompanying this questionnaire. On the next page, 24 definitions are listed (most of them are found in the reader).

Question 10: Read the definitions and score the eight definitions that you find most closely resemble the concept of vulnerability with 2; score the eight definitions that resemble the concept of vulnerability less so with 1; score the eight definitions that you find least fitting with 0. A small shift in proportion is acceptable.

The 10 highest ranking definitions will be discussed on the meeting. Please write a 0, 1, or 2 behind each statement.

	Definition vulnerability	Judgement 0 / 1 / 2
1	Frailty is a dynamic state affecting an individual who experiences losses in one or more domains of human functioning (physical, psychological, social), which is caused by the influence of a range of variables and which increases the risk of adverse outcomes (Gobbens, 2010).	
2	Vulnerable populations are populations at risk for poor physical, psychological, and/or social health (Aday, 1994).	
3	Vulnerability is the propensity of social or ecological systems to suffer harm from external stresses and perturbations (Kasperson et al, 1995 in deFur et al, 2007).	
4	Vulnerability is a multidimensional construct reflecting a convergence of many risk factors at both the individual and community levels, which influence health and healthcare experiences (Shi et al, 2005 in Shi et al, 2008).	
5	Vulnerable groups are social groups who have an increased relative risk or susceptibility to adverse health outcomes (Flaskerud, 1998 in Flaskerud et al, 1998).	
6	Vulnerability is defined as the constellation of past, present and future risk, perceived or real, as a result of the common human experience of risk, the increased vulnerability of the adolescent period, consequences of family disruption, and increased risks of life on the street (Dorsen, 2010).	
7	Vulnerability is the susceptibility to harm resulting from the interaction of risk factors and supports and resources available to individuals and groups (Mechanic et al, 2007).	
8	Vulnerability is an increased susceptibility to health and health care disparities due to a combination of individual and environmental factors (Grabovschi et al, 2013).	
9	Frailty is an accumulation of deficits across physical, psychological, and social domains (Salem et al, 2014).	
10	Vulnerability is a condition of heightened fragility of a population or specific group, and a process that is potentially reversible or avoidable through appropriate interventions (Zarowsky et al, 2013).	
11	Vulnerability is the progressive loss of wellbeing, i.e. health, related to social and economic deprivation (WHO definition in Allotey et al, 2012 in Zarowsky et al, 2013).	
12	Vulnerable populations are groups that are clinically at risk and/or socially disadvantaged (Lewis et al, 2012).	
13	Vulnerability is the universally present relative risk of potential or actual harm from external judgments of endangerment, functional capacity, and socially sanctioned need for intervention (Demi et al, 1995 in Spiers, 2000).	
14	Vulnerability is the experience of exposure to harm which challenge one's integrity (Spiers, 2000).	
15	Vulnerable groups are social groups who experience limited resources and consequent high relative risk for morbidity and premature mortality (London, 2007 in Amin et al, 2011).	
16	Social vulnerability is a precarious economic situation justifying the allocation of welfare benefits and/or resulting in inadequate health coverage (Marmot, 2005 and Ridde, 2007 in Pascal et al, 2009).	
17	To be vulnerable means to face a significant probability of incurring an identifiable harm while substantially lacking ability and/or means to protect oneself (Schroeder et al, 2009).	
18	To be vulnerable means to be substantially incapable of protecting one's own interests (Council for International Organizations of Medical Science [CIOMS] in Schroeder et al, 2009).	
19	Vulnerable populations are groups at increased risk for poor physical, psychological, and social health outcomes and inadequate health care (Aday, 2001 and Flaskerud et al, 1998 in Pitkin Derose et al, 2007).	
20	Vulnerable populations are those at greater risk for poor health status and health care access (Shi et al, 2004).	
21	Vulnerability speaks to susceptibility to health problems, harm, or neglect (Phillips, 1992 en Rogers, 1997 in Bragg Leight, 2003).	
22	Vulnerable populations are populations in which complex medical needs are exacerbated by social needs (Vanderbilt et al, 2013).	
23	Vulnerable people are those who are less likely than average to obtain medical care of an appropriate quality and quantity (Pauly et al, 2007).	
24	Vulnerable populations are groups, whose demographic, geographic, or economic characteristics impede or prevent their access to health care services (Blumentahal et al, 1995).	

Task 6: Vulnerability: how do you determine a client's vulnerability status?

This task is about the best methods to measure/document the vulnerability (presence, degree of) of a client, person or family (not: neighborhood, or other geographical area).
Vulnerability can be determined using a self-report questionnaire, in an interview with a care professional, or retrospectively drawn from administrative sources (civil or clinical registry).

Question 11: In the different contexts below, how can the vulnerability status of a client, person or family best be determined?

	Psychiatry	Elderly	Pregnant women	Child services	Chronically ill
Questionnaire / checklist professional support					
Questionnaire / checklist self-report					
Face to face during consult					
Registry (e.g. medical data)					

Question 12: Give an example of 'best practice' for determining an individual client's vulnerability (preferably own observation/experience).

Answer:

You have reached the end of the questionnaire.
Thank you for filling it out.

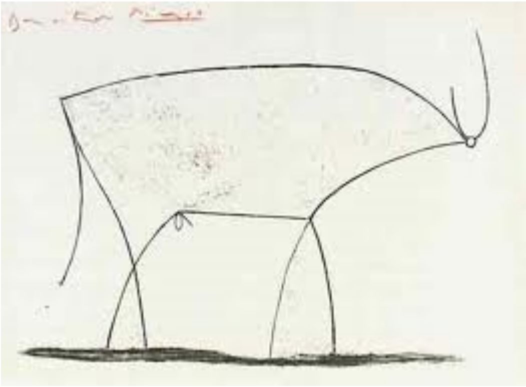

Supplement: S2 Appendix — (PDF) [file pone.0212633.s002.pdf]
