# Supplementary material for: Towards a universal concept of vulnerability: Broadening the evidence from the elderly to perinatal health using a Delphi approach
Source: PLoS One. 2019 Feb 20;14(2):e0212633. doi: 10.1371/journal.pone.0212633 (PMC6382270; doi:10.1371/journal.pone.0212633)
Supplement: S3 Appendix — (PDF) [file pone.0212633.s003.pdf]

1. **Population**

('vulnerable population'/de OR 'high risk population'/de OR 'high risk patient'/de OR 'frail elderly'/de OR frail* OR vulnerab* ((vulnerab* OR risk* OR disadvantage* OR inequal* OR unequal* OR inequit* OR depriv*) NEAR/3(people OR person OR population OR group OR patient)):ab,ti)

2. **Pregnancy**

('pregnancy'/exp OR 'adolescent pregnancy'/de OR 'first trimester pregnancy'/de OR 'multiple pregnancy'/de OR 'twin pregnancy'/de OR 'second trimester pregnancy'/de OR 'third trimester pregnancy'/de OR 'unplanned pregnancy'/de OR 'unwanted pregnancy'/de OR ((pregnan*) NEAR/3 (adolescen* OR teen* OR multip* OR twin* OR quad* OR quin* OR trip* OR trimester OR early OR late OR unplanned OR unintended OR unwanted OR undesired OR intrauterine OR maintenance)) OR twinning* OR 'multiple birth' OR quadruplet* OR quintuplet* OR triplet* OR superfetation OR 'child bearing' OR childbearing OR gestation OR gravidity OR 'labor presentation' OR ((trimester) NEAR/3 (first OR second OR third OR 1st OR 2nd OR 3rd OR mid*)):ab,ti)

3. **PROGRESS**

('neighborhood'/de OR 'community'/de OR 'religion'/de OR 'occupation'/de OR 'employment'/exp OR 'income'/de OR 'ethnicity'/de OR 'race'/de OR 'education'/de OR 'social status'/exp OR 'lowest income group'/de OR 'poverty'/de OR 'social background'/de OR 'social class'/de OR 'social network'/de OR 'social capital'/de OR 'health literacy'/de OR 'locus of control'/de OR 'attitude to health'/de OR 'health insurance'/de OR 'private insurance'/de OR 'public health insurance'/de OR 'social stigma'/de OR 'stigma'/de OR 'risk perception' OR 'medically uninsured'/de OR 'risk factor'/de OR 'relative risk' OR ((health*)NEAR/3(knowledge OR attitude* OR practice OR accept* OR barrier)) OR 'HLC' OR 'health locus of control' OR uninsured OR 'insurance status' OR neighborhood OR neighbourhood OR 'poverty areas' OR 'religious belief' OR spiritual* OR profession OR 'employment status' OR income OR 'social network' OR 'sociocultural class' OR 'socioeconomic class' OR residenc* OR

((education*)NEAR/3(achievement OR attain* OR level)) OR ‘social determinants of health’/de OR ((social*)NEAR/3(determin* OR factor)) OR ((underserve*) NEAR/3(neighborhood OR neighbourhood OR area)) OR ((social* OR socio* OR religious OR gender OR politic* OR econom*) NEAR/3 (barrier OR threshold)):ab,ti)

4. **Health care outcome**

(‘health care system’/exp OR ‘health care access’/de OR ‘health care availability’/de OR ‘health care disparity’/de OR ‘health care distribution’/de OR ‘health care need’/de OR ‘health care planning’/de OR ‘healthcare system’/de OR ‘health disparity’/de OR ‘health care delivery’/de OR ‘ambulatory care’/de OR ‘hospital care’/de OR ‘primary health care’/de OR ‘secondary health care’/de OR ‘tertiary care center’/de OR ‘health care quality’/de OR ((health* OR service*) NEAR/3(access* OR availab* OR disparat* OR equit* OR inequal* OR plan* OR priorit* OR resource* OR suppl* OR deliver*)):ab,ti)

5. **Pregnancy outcome**

(‘maternal morbidity’/de OR ‘maternal mortality’/de OR ‘perinatal death’/de OR ‘perinatal morbidity’/exp OR ‘newborn morbidity’/de OR ‘newborn death’/de OR ‘child death’/de OR ‘infant mortality’/de OR ‘small for date infant’/de OR ‘low birth weight’/exp OR ‘extremely low birth weight’/de OR ‘very low birth weight’/de OR ‘apgar score’/de OR ((maternal OR mother OR perinatal OR neonat* OR infant*) NEAR/3 (death OR mortality OR morbidity OR disease)) OR ‘small for gestational age’ OR ((SGA OR ‘small for gestational age’ OR ‘small for date’ OR ‘small for age’) NEAR/3 (infant* OR neonat* OR newborn OR baby)) OR ‘ELBW’ OR ‘VLBW’ OR ‘LBW’ OR ((‘birth weight’ OR birthweight OR ‘LBW’ OR underweight) NEAR/3 (infant* OR neonat* OR newborn OR low)) OR ((apgar OR APGAR) NEAR/3 (classif* OR coeff* OR index* OR rating OR scale OR test OR timer OR score)):ab,ti
