# Supplementary material for: Towards a universal concept of vulnerability: Broadening the evidence from the elderly to perinatal health using a Delphi approach
Source: PLoS One. 2019 Feb 20;14(2):e0212633. doi: 10.1371/journal.pone.0212633 (PMC6382270; doi:10.1371/journal.pone.0212633)
Supplement: S1 Table — (PDF) [file pone.0212633.s004.pdf]

Nr	Title	Group	Vulnerability pathway
1	In search of an Integral Conceptual Definition of Frailty: Opinions of Experts [1]	The elderly	Pathway to unhealthy
2	Health Status of Vulnerable Populations [2]	Generic	Pathway to unhealthy
3	Vulnerability as a Function of Individual and Group Resources in Cumulative Risk Assessment [3]	Generic	Pathway to unhealthy
4	Rethinking Vulnerable Populations in the United States: An Introduction to a General Model of Vulnerability [4]	Generic	Pathway to unhealthy & Pathway to healthy
5	Enhancing the Measurement of Health Disparities for Vulnerable Populations [5]	Generic	Pathway to unhealthy & Pathway to healthy
6	Vulnerability, health and health care [6]	Generic	Pathway to healthy
7	Conceptualizing Vulnerable Populations Health-Related Research [7]	Generic	Pathway to unhealthy
8	Vulnerability in homeless adolescents: concept analysis [8]	The homeless	Pathway to unhealthy
9	Vulnerable People, Groups, And Populations: Societal View [9]	Generic	Pathway to unhealthy
10	Mapping the concept of vulnerability related to health care disparities: a scoping review [10]	Generic	Pathway to unhealthy & Pathway to healthy
11	The comprehensive frailty assessment instrument: Development, validity and reliability [11]	The elderly	Pathway to unhealthy
12	Construction and identifying predictors of frailty among homeless adults - A latent variable structural equations model approach [12]	The homeless	Pathway to unhealthy
13	Beyond 'vulnerable groups': contexts and dynamics of vulnerability [13]	Generic	Pathway to unhealthy
14	The Promise and Peril of Accountable Care for Vulnerable Populations: a Framework for Overcoming Obstacles [14]	Generic	Pathway to healthy
15	New perspectives on vulnerability using emic and etic approaches [15]	Generic	Pathway to unhealthy
16	Epidemiology of multimorbidity and implications for health care, research, and medical education: a cross-sectional study [16]	Generic	Pathway to unhealthy
17	EquiFrame: A framework for analysis of the inclusion of human rights and vulnerable groups in health policies [17]	Generic	Pathway to unhealthy
18	The Vulnerability of Middle-Aged and Older Adults in a Multiethnic, Low-Income Area: Contributions of Age, Ethnicity, and Health Insurance [18]	The elderly	Pathway to healthy
19	Social vulnerability and unmet preventive care needs in outpatients of two French public hospitals [19]	Outpatients	Pathway to healthy
20	Vulnerability: Too Vague and Too Broad? [20]	Generic	Pathway to unhealthy
21	Immigrants and Health Care: Sources of Vulnerability [21]	Immigrants	Pathway to unhealthy
22	Access to Care, Health Status, and Health Disparities in the United States and Canada: Results of a Cross-National Population-Based Survey [22]	Generic	Pathway to healthy
23	Vulnerability and Unmet Health Care Needs. The Influence of Multiple Risk Factors [23]	Generic	Pathway to unhealthy & Pathway to healthy

24	The Application of a Vulnerable Populations Conceptual Model to Rural Health [24]	Rural populations	Pathway to unhealthy
25	Towards an integral conceptual model of frailty [25]	The elderly	Pathway to unhealthy
26	Psychometric Properties of the Dutch Version of the Self-Sufficiency Matrix (SSM-D) [26]	Generic	Pathway to healthy
27	Inequalities in perinatal and maternal health [27]	Pregnant women	Pathway to unhealthy
28	An innovative screen-and-advice model for psychopathology and psychosocial problems among urban pregnant women: an exploratory study [28]	Pregnant women	Pathway to unhealthy
29	Patient Versus Professional Based Psychosocial Risk Factor Screening for Adverse Pregnancy Outcomes [29]	Pregnant women	Pathway to unhealthy

References

1. Gobbens RJ, Luijkx KG, Wijnen-Sponselee MT, Schols JM. In search of an integral conceptual definition of frailty: opinions of experts. *J Am Med Dir Assoc.* 2010;11: 338-343.
2. Aday, LA. Health status of vulnerable populations. *Annu Rev Public Health.* 1994;15: 487-509.
3. DeFur PL, Evans GW, Cohen Hubal EA, Kyle AD, Morello-Frosch RA, Williams DR. Vulnerability as a function of individual and group resources in cumulative risk assessment. *Environ Health Perspect.* 2007;115: 817-824.
4. Shi L, Stevens GD, Faed P, Tsai J. Rethinking vulnerable populations in the United States: an introduction to a general model of vulnerability. *Harvard Health Policy Review.* 2008;9: 43-48.
5. Shi L, Stevens GD, Lebrun LA, Faed P, Tsai J. Enhancing the measurement of health disparities for vulnerable populations. *J Public Health Man.* 2008;14: S45-S53.
6. Rogers AC. Vulnerability, health and health care. *J Adv Nurs.* 1997;26: 65-72.
7. Flaskerud JH, Winslow BJ. Conceptualizing vulnerable populations health-related research. *Nurs Res.* 1998;47: 69-78.
8. Dorsen C. Vulnerability in homeless adolescents: concept analysis. *J Adv Nurs.* 2010;66: 2819-2827.
9. Mechanic D, Tanner J. Vulnerable people, groups, and populations: societal view. *Health Affair.* 2007;26: 1220-1230.
10. Grabovschi C, Loignon C, Fortin M. Mapping the concept of vulnerability related to health care disparities: a scoping review. *BMC Health Serv Res.* 2013;94: 1-11.
11. de Witte N, Gobbens R, De Donder L, Dury S, Buffel T, Schols J, et al. The comprehensive frailty assessment instrument: development, validity and reliability. *Geriatr Nurs.* 2013;34: 274-281.
12. Salem BE, Nyamathi A, Brecht ML, Phillips LR, Mentes JC, Sarkisian C, et al. Constructing and identifying predictors of frailty among homeless adults – a latent variable structural equations model approach. *Arch Gerontol Geriat.* 2014;58: 248-256.

13. Zarowsky C, Haddad S, Nguyen V., Beyond ‘vulnerable groups’: contexts and dynamics of vulnerability. *Global Health Promotion.* 2013;20(Supp 1): 3-9.
14. Lewis VA, Larson BK, McClurg AB, Boswell RG, Fisher ES. The promise and peril of accountable care for vulnerable populations: a framework for overcoming obstacles. *Health Affair.* 2012;31: 1777-1785.
15. Spiers J. New perspectives on vulnerability using emic and etic approaches. *J Adv Nurs.* 2000;31: 715-721.
16. Barnett K, Mercer SW, Norbury M, Watt G, Wyke S, Guthrie B. Epidemiology of multimorbidity and implications for health care, research, and medical education: a cross-sectional study. *Lancet.* 2012;380; 37-43.
17. Amin M, MacLachlan M, Mannan H, El Tayeb S, El Khatim A, Swartz L, et al. EquiFrame: a framework for analysis of the inclusion of human rights and vulnerable groups in health policies. *Health and Human Rights.* 2011;13: 1-20.
18. Walker KO, Steers N, Liang LJ, Morales LS, Forge N, Jones L, et al. The vulnerability of middle-aged and older adults in a multiethnic, low-income area: contributions of age, ethnicity, and health insurance. *J Am Geriatr Soc.* 2010;58: 2416-2422.
19. Pascal J, Abbey-Huguenin H, Leux C, Lombrail P, Lert F. Social vulnerability and unmet preventive care needs in outpatients of two French public hospitals. *Eur J Public Health.* 2009;19: 403-411.
20. Schroeder D, Gefenas E. Vulnerability: too vague and too broad? *Camb Q Healthc Ethic.* 2009;18: 113-121.
21. Derose KP, Escarce JJ, Lurie N. Immigrants and health care: sources of vulnerability. *Health Affair.* 2007;26: 1258-1268.
22. Lasser KE, Himmelstein DU, Woolhandler S. Access to care, health status, and health disparities in the United States and Canada: results of a cross-national population-based survey. *Am J Public Health.* 2006;96: 1300-1307.
23. Shi L, Stevens GD. Vulnerability and unmet health care needs. The influence of multiple risk factors. *J Gen Intern Med.* 2005;20: 148-154.
24. Leight SB. The application of a vulnerable populations conceptual model to rural health. *Public Health Nurs.* 2003;20: 440-448.
25. Gobbens RJJ, Luijkx KG, Wijnen-Sponselee MT, Schols JMGA. Towards an integral conceptual model of frailty. *J Nutr Health Aging.* 2010;14: 175-181.
26. Fassaert T, Lauriks S, Van de Weerd S, Theunissen J, Kikkert M, Dekker J, et al. Psychometric properties of the Dutch version of the self-sufficiency matrix (SSM-D). *Community Ment Hlt J.* 2014;50: 583-590.
27. de Graaf JP, Steegers EA, Bonsel GJ. Inequalities in perinatal and maternal health. *Curr Opin Obstet Gynecol.* 2013;25: 98-108.
28. Quispel C, Schneider TAJ, Bonsel GJ, Lambregtse-van den Berg MP. An innovative screen-and-advice model psychopathology and psychosocial problems among urban pregnant women: an exploratory study. *J Psychosom Obst Gyn*. 2012;33: 7-14.
29. Quispel C, van Veen MJ, Zuijderhoudt C, Steegers EAP, Hoogendijk WJG, Birnie E, et al. Patient versus professional based psychosocial risk factor screening for adverse pregancy outcomes. *Matern Child Healt J.* 2014;18: 2089-2097.
