# Supplementary material for: Towards a universal concept of vulnerability: Broadening the evidence from the elderly to perinatal health using a Delphi approach
Source: PLoS One. 2019 Feb 20;14(2):e0212633. doi: 10.1371/journal.pone.0212633 (PMC6382270; doi:10.1371/journal.pone.0212633)
Supplement: S2 Table — (PDF) [file pone.0212633.s005.pdf]

Nr	Elements of vulnerability
1	Age (low and high)
2	Female gender
3	High exposure to risks
4	High risk occupation
5	Insufficient coping
6	Lack of ability to take responsibility for one's health
7	Lack of insurance coverage
8	Lack of material resources
9	Lack of motivation
10	Lack of reserve capacity
11	Lack of resilience
12	Living in a deprived neighborhood
13	Low (preventive) health care accessibility and quality
14	Low education
15	Low income / poverty
16	Low sense of control and mastery
17	Low social status
18	Low social support
19	Minority race / ethnicity
20	Negative perception of situation
21	Poor physical health
22	Poor psychological health
23	Psychosocial stress
24	Religion
25	Small social network
26	Stigma
27	Substance abuse
28	Unhealthy activities and behaviors
29	Accumulation of risks
