# Supplementary material for: Towards a universal concept of vulnerability: Broadening the evidence from the elderly to perinatal health using a Delphi approach
Source: PLoS One. 2019 Feb 20;14(2):e0212633. doi: 10.1371/journal.pone.0212633 (PMC6382270; doi:10.1371/journal.pone.0212633)
Supplement: S3 Table — (PDF) [file pone.0212633.s006.pdf]

Nr	Definition of vulnerability
1	Frailty is a dynamic state affecting an individual who experiences losses in one or more domains of human functioning (physical, psychological, social), which is caused by the influence of a range of variables and which increases the risk of adverse outcomes [1].
2	Vulnerable populations are populations at risk for poor physical, psychological, and/or social health [2].
3	Vulnerability is the propensity of social or ecological systems to suffer harm from external stresses and perturbations [3].
4	Vulnerability is a multidimensional construct reflecting a convergence of many risk factors at both the individual and community levels, which influence health and healthcare experiences [4].
5	Vulnerable groups are social groups who have an increased relative risk or susceptibility to adverse health outcomes [5].
6	Vulnerability is defined as the constellation of past, present and future risk, perceived or real, as a result of the common human experience of risk, the increased vulnerability of the adolescent period, consequences of family disruption, and increased risks of life on the street [6].
7	Vulnerability is the susceptibility to harm resulting from the interaction of risk factors and supports and resources available to individuals and groups [7].
8	Vulnerability is an increased susceptibility to health and health care disparities due to a combination of individual and environmental factors [8].
9	Frailty is an accumulation of deficits across physical, psychological, and social domains [9].
10	Vulnerability is a condition of heightened fragility of a population or specific group, and a process that is potentially reversible or avoidable through appropriate interventions [10].
11	Vulnerability is the progressive loss of wellbeing, i.e. health, related to social and economic deprivation [10].
12	Vulnerable populations are groups that are clinically at risk and/or socially disadvantaged [11].
13	Vulnerability is the universally present relative risk of potential or actual harm from external judgments of endangerment, functional capacity, and socially sanctioned need for intervention [12].
14	Vulnerability is the experience of exposure to harm which challenge one's integrity [12].
15	Vulnerable groups are social groups who experience limited resources and consequent high relative risk for morbidity and premature mortality [13].
16	Social vulnerability is a precarious economic situation justifying the allocation of welfare benefits and/or resulting in inadequate health coverage [14].
17	To be vulnerable means to face a significant probability of incurring an identifiable harm while substantially lacking ability and/or means to protect oneself [15].
18	To be vulnerable means to be substantially incapable of protecting one's own interests [15].
19	Vulnerable populations are groups at increased risk for poor physical, psychological, and social health outcomes and inadequate health care [16].
20	Vulnerable populations are those at greater risk for poor health status and health care access [17].
21	Vulnerability speaks to susceptibility to health problems, harm, or neglect [18].
22	Vulnerable populations are populations in which complex medical needs are exacerbated by social needs [19].
23	Vulnerable people are those who are less likely than average to obtain medical care of an appropriate quality and quantity [20].
24	Vulnerable populations are groups, whose demographic, geographic, or economic characteristics impede or prevent their access to health care services [21].

References

1. Gobbens RJ, Luijkx KG, Wijnen-Sponselee MT, Schols JM. In search of an integral conceptual definition of frailty: opinions of experts. *J Am Med Dir Assoc.* 2010;11: 338-343.
2. Aday, LA. Health status of vulnerable populations. *Annu Rev Public Health.* 1994;15: 487-509.
3. DeFur PL, Evans GW, Cohen Hubal EA, Kyle AD, Morello-Frosch RA, Williams DR. Vulnerability as a function of individual and group resources in cumulative risk assessment. *Environ Health Perspect.* 2007;115: 817-824.
4. Shi L, Stevens GD, Lebrun LA, Faed P, Tsai J. Enhancing the measurement of health disparities for vulnerable populations. *J Public Health Man.* 2008;14: S45-S53.
5. Flaskerud JH, Winslow BJ. Conceptualizing vulnerable populations health-related research. *Nurs Res.* 1998;47: 69-78.
6. Dorsen C. Vulnerability in homeless adolescents: concept analysis. *J Adv Nurs.* 2010;66: 2819-2827.
7. Mechanic D, Tanner J. Vulnerable people, groups, and populations: societal view. *Health Affair.* 2007;26: 1220-1230.
8. Grabovschi C, Loignon C, Fortin M. Mapping the concept of vulnerability related to health care disparities: a scoping review. *BMC Health Serv Res.* 2013;94: 1-11.
9. Salem BE, Nyamathi A, Brecht ML, Phillips LR, Mentes JC, Sarkisian C, et al. Constructing and identifying predictors of frailty among homeless adults – a latent variable structural equations model approach. *Arch Gerontol Geriat.* 2014;58: 248-256.
10. Zarowsky C, Haddad S, Nguyen V., Beyond ‘vulnerable groups’: contexts and dynamics of vulnerability. *Global Health Promotion.* 2013;20(Supp 1): 3-9.
11. Lewis VA, Larson BK, McClurg AB, Boswell RG, Fisher ES. The promise and peril of accountable care for vulnerable populations: a framework for overcoming obstacles. *Health Affair.* 2012;31: 1777-1785.
12. Spiers J. New perspectives on vulnerability using emic and etic approaches. *J Adv Nurs.* 2000;31: 715-721.
13. Amin M, MacLachlan M, Mannan H, El Tayeb S, El Khatim A, Swartz L, et al. EquiFrame: a framework for analysis of the inclusion of human rights and vulnerable groups in health policies. *Health and Human Rights.* 2011;13: 1-20.
14. Pascal J, Abbey-Huguenin H, Leux C, Lombrail P, Lert F. Social vulnerability and unmet preventive care needs in outpatients of two French public hospitals. *Eur J Public Health.* 2009;19: 403-411.

15. Schroeder D, Gefenas E. Vulnerability: too vague and too broad? *Camb Q Healthc Ethic.* 2009;18: 113-121.
16. Derose KP, Escarce JJ, Lurie N. Immigrants and health care: sources of vulnerability. *Health Affair.* 2007;26: 1258-1268.
17. Shi L, Stevens GD. Vulnerability and unmet health care needs. The influence of multiple risk factors. *J Gen Intern Med.* 2005;20: 148-154.
18. Leight SB. The application of a vulnerable populations conceptual model to rural health. *Public Health Nurs.* 2003;20: 440-448.
19. Vanderbilt AA, Isringhausen KT, VanderWielen LM, Wright MS, Slashcheva LD, Madden MA. Health disparities among highly vulnerable populations in the United States: a call to action for medical and oral health care. *Med Educ Online.* 2013;18: 1-3.
20. Pauly MV, Pagan JA. Spillovers and vulnerability: the case of community uninsurance. *Health Affair.* 2007;26: 1304-1314.
21. Blumenthal D, Mort E, Edwards J. The efficacy of primary care for vulnerable population groups. *Health Serv Res.* 1995;30: 253-273.
